# Time-dependent heterogeneity leads to transient suppression of the COVID-19 epidemic, not herd immunity

**DOI:** 10.1073/pnas.2015972118

**Published:** 2021-04-08

**Authors:** Alexei V. Tkachenko, Sergei Maslov, Ahmed Elbanna, George N. Wong, Zachary J. Weiner, Nigel Goldenfeld

**Affiliations:** ^a^Center for Functional Nanomaterials, Brookhaven National Laboratory, Upton, NY 11973;; ^b^Department of Physics, University of Illinois at Urbana–Champaign, Urbana, IL 61801;; ^c^Department of Bioengineering, University of Illinois at Urbana–Champaign, Urbana, IL 61801;; ^d^Carl R. Woese Institute for Genomic Biology, University of Illinois at Urbana–Champaign, Urbana, IL 61801;; ^e^Department of Civil Engineering, University of Illinois at Urbana–Champaign, Urbana, IL 61801;; ^f^Beckman Institute for Advanced Science and Technology, University of Illinois at Urbana-Champaign, Urbana, IL 61801

**Keywords:** COVID-19, heterogeneity, overdispersion, epidemic theory

## Abstract

Epidemics generally spread through a succession of waves that reflect factors on multiple timescales. On short timescales, superspreading events lead to burstiness and overdispersion, whereas long-term persistent heterogeneity in susceptibility is expected to lead to a reduction in both the infection peak and the herd immunity threshold (HIT). Here, we develop a general approach to encompass both timescales, including time variations in individual social activity, and demonstrate how to incorporate them phenomenologically into a wide class of epidemiological models through reparameterization. We derive a nonlinear dependence of the effective reproduction number $R_{e}$ on the susceptible population fraction S. We show that a state of transient collective immunity (TCI) emerges well below the HIT during early, high-paced stages of the epidemic. However, this is a fragile state that wanes over time due to changing levels of social activity, and so the infection peak is not an indication of long-lasting herd immunity: Subsequent waves may emerge due to behavioral changes in the population, driven by, for example, seasonal factors. Transient and long-term levels of heterogeneity are estimated using empirical data from the COVID-19 epidemic and from real-life face-to-face contact networks. These results suggest that the hardest hit areas, such as New York City, have achieved TCI following the first wave of the epidemic, but likely remain below the long-term HIT. Thus, in contrast to some previous claims, these regions can still experience subsequent waves.

## Significance

Epidemics generally spread through a succession of waves that reflect factors on multiple timescales. Here, we develop a general approach bridging across these timescales and demonstrate how to incorporate population heterogeneity into a wide class of epidemiological models. We demonstrate that a fragile state of transient collective immunity emerges during early, high-paced stages of the epidemic, leading to suppression of individual epidemic waves. However, this state is not an indication of lasting herd immunity: Subsequent waves may emerge due to stochastic changes in individual social activity. Parameters of transient collective immunity are estimated using empirical data from the COVID-19 epidemic in several US locations.

The COVID-19 pandemic is nearly unprecedented in the level of disruption it has caused globally, but also, potentially, in the degree to which it will change our understanding of epidemic dynamics and the efficacy of various mitigation strategies. Ever since the pioneering works of Kermack and McKendrick (1), epidemiological models have been widely and successfully used to quantify and predict the progression of infectious diseases (2–6). More recently, the important role in epidemic spreading played by population heterogeneity and the complex structure of social networks has been appreciated and highlighted in multiple studies (7–25). However, integration of this conceptual progress into reliable, predictive epidemiological models remains a formidable task. Among the key effects of heterogeneity and social network structure are 1) the role played by superspreaders and superspreading events during initial outbreaks (12, 13, 16, 26–29) and 2) a substantial reduction of the final size of epidemic (FSE) as well as the herd immunity threshold (HIT) compared to the homogeneous case (9, 10, 19–21, 23, 30). The COVID-19 pandemic has reignited interest in the effects of heterogeneity of individual susceptibility to the disease, in particular, to the possibility that it might lower both HIT and FSE (31–35). In studying epidemics in heterogeneous populations, it is important to emphasize the qualitative nature of two important timescales. First, overdispersion is dominated by short-term patterns of behavior and even accidental events rather than persistent population behavioral heterogeneity. Second, short-term overdispersion is generally assumed to have a limited impact on the long-term epidemic dynamics, being important primarily during early outbreaks dominated by superspreading events. In this paper, we attempt to provide a multiscale theory for epidemic progression and show that both overdispersion and persistent heterogeneity affect the overall progression of the COVID-19 epidemic. The significance of this multiscale perspective is that it provides a natural formalism to predict the occurrence and nature of successive epidemic waves, even when it might seem that a first wave has attained a state which could be mistaken for herd immunity.

There are several existing approaches to model the effects of heterogeneity on epidemic dynamics, each focusing on a different characteristic and parameterization. In the first approach, one stratifies the population into several demographic groups (e.g., by age), and accounts for variation in susceptibility of these groups and their mutual contact probabilities (2). While this approach represents many aspects of population dynamics beyond the homogeneous and well-mixed assumption, it clearly does not encompass the whole complexity of individual heterogeneity, interpersonal communications, and spatial and social structures. These details can be addressed in a second approach, where one analyzes the epidemic dynamics on real-world or artificial social networks (8, 13, 23, 36, 37). Through elegant mathematics, it is possible to obtain detailed results in idealized cases, including the mapping onto well-understood models of statistical physics such as percolation (9, 38). As demonstrated in ref. 21, the FSE is a very robust property of the epidemic, insensitive to fine details of its dynamics (39) defined by 1) distribution of susceptibilities in the population (20, 30, 40) and 2) correlations between infectivity and susceptibility. Importantly, it does not depend on the part of heterogeneous infectivity that is not correlated with susceptibility. However, these approaches, so far, have been mostly limited to the analysis of the FSE and distribution of outbreak sizes on a static social network.

For practical purposes, it is desirable to predict the complete time-dependent dynamics of an epidemic, preferably by explicitly including heterogeneity into classical well-mixed mean-field compartmentalized models. This approach was developed some time ago in the context of epidemics on networks (10, 23), and the resulting mean-field theory effectively reproduces the structure of heterogeneous well-mixed models extensively studied in the applied mathematics literature (19–22, 24, 30). The overall impact of heterogeneity occurs because, as the disease spreads, it preferentially immunizes the more susceptible individuals, so the remaining population is less susceptible, and spread is inhibited. This effect is further enhanced by a positive correlation between infectivity and susceptibility. In the context of static network models, this correlation is perfect, since both factors are proportional to the degree of individual nodes. Ref. 24 studied a hybrid model in which social heterogeneity represented by network degree was combined with a biological one. These approaches have been recently applied in the context of COVID-19 (31, 32, 35, 41, 42). The conclusion of these studies was that the HIT may be well below that expected in classical homogeneous models.

These approaches to heterogeneity delineate end-members of a continuum of theories: overdispersion describing short-term, bursty dynamics (e.g., due to superspreader accidents), as opposed to *persistent heterogeneity*, which is a long-term characteristic of an individual and reflects behavioral propensity, for example, to socialize in large gatherings without prudent social distancing. Overdispersion is usually modeled in terms of a negative binomial branching process (12, 13, 16, 26–28). Strictly speaking, both persistent heterogeneity and short-term variations contribute to the overdispersion of individual reproduction number. However, we will see below that the former is likely to be a much weaker source of variation compared to the latter. It is also generally presumed that short-term overdispersion is uncorrelated in time and thus has no effect on epidemic dynamics. Indeed, large variations in an individual’s infectivity would average out as long as they are not correlated with susceptibility. But, since the initial exposure and secondary infections are separated by a single generation interval (typically about 5 d for COVID-19), the levels of individual social activity at those times are expected to be correlated, and (at least partially) reflect short-term overdispersion. How, then, can one understand the epidemic progression across various timescales, from early stages of a fast exponential growth to the final state of the herd immunity?

Below, we present a comprehensive yet simple theory that accounts for both social and biological aspects of heterogeneity, and predicts how, together, they modify early and intermediate epidemic dynamics, as well as global characteristics of the epidemic such as its HIT. Importantly, early epidemic dynamics may be sensitive to both persistent heterogeneity and short-term overdispersion. As a result, the apparent early-stage heterogeneity is typically enhanced compared to its long-term persistent level. This may lead to a suppression of the first wave of the epidemic due to reaching a novel state that we call transient collective immunity (TCI) determined by a combination of short-term and long-term heterogeneity, whose threshold is lower than the eventual HIT. The implication is that the first wave of an epidemic ends due to a combination of both persistent heterogeneity and whatever mitigation constraints are imposed on the population. As the latter are relaxed by authorities or through behavioral changes associated with seasonal factors, subsequent waves can still occur. Thus, TCI is dramatically different from the idea of herd immunity due to heterogeneity.

Our starting point is a generalized version of the heterogeneous well-mixed theory in the spirit of ref. 10, but we use the age-of-infection approach (1) rather than compartmentalized susceptible, infectious, recovered (SIR)/susceptible, exposed, infectious, recovered (SEIR) models of epidemic dynamics (see, e.g., ref. 2). Similar to multiple previous studies, we first completely ignore any time dependence of individual susceptibilities and infectivities, focusing only on their long-term persistent components. This approach implicitly assumes that any short-term overdispersion (responsible for, e.g., the superspreading phenomenon) is uncorrelated in time and thus effectively averaged out. This is a valid assumption if the large short-term variations in individual infectivity are completely uncorrelated with an individual’s susceptibility. However, this approximation is hard to justify in the case of COVID-19. Indeed, if the two are correlated on the timescale of a single generation interval (5 d), this will strongly affect the overall epidemic dynamics. Therefore, our initial analysis is eventually expanded to a more general theory accounting for the nonnegligible effects of short-term overdispersion. In the case of persistent heterogeneity, we demonstrate how the model can be recast into an effective homogeneous theory that can readily encompass a wide class of epidemiological models, including various versions of the popular SIR/SEIR approaches. Specific innovations that emerge from our analysis are the nonlinear dependence of the effective reproduction number $R_{e}$ on the overall population fraction S of susceptible individuals, and another nonlinear function $S_{e}$ that gives an effective susceptible fraction, taking into account preferential removal of highly susceptible individuals.

A convenient and practically useful aspect of this approach is that it does not require extensive additional calibration in order to be applied to real data. In the effort to make quantitative predictions from epidemic models, accurate calibration is arguably the most difficult step, but is necessary due to the extreme instability of epidemic dynamics in both growth and decay phases (43, 44). We find that, with our approach, the entire effect of heterogeneity is, in many cases, well characterized by a single parameter which we call the *immunity factor* λ. It is related to the statistical properties of heterogeneous susceptibility across the population and to its correlation with individual infectivity. The immunity factor determines the rate at which $R_{e}$ drops during the early stages of the epidemic as the pool of susceptibles is being depleted: $R_{e}\approx R_{0}\left(1-\lambda \left(1-S\right)\right)$. Beyond this early linear regime, for an important case of gamma-distributed individual susceptibilities, we show that the classical proportionality, $R_{e}=R_{0}S$, transforms into a power law scaling relationship $R_{e}=R_{0}S^{\lambda }$. This leads to a modified version of the result for the HIT, $1-S_{0}=1-R_{0}^{-1/\lambda }$.

Heterogeneity in the susceptibility of individual members of the population has several different contributions: 1) biological, which takes into account differences in factors such as strength of immune response, genetics, age, and comorbidities; and 2) social, reflecting differences in the number and frequency of close contacts of different people. The immunity factor λ in our model combines these sources of heterogeneous susceptibility as well as its correlation with individual infectivity. As we demonstrate, under certain assumptions, the immunity factor is simply a product of social and biological contributions: $\lambda =\lambda_{s}\lambda_{b}$. In our study, we leverage existing studies of real-life face-to-face contact networks (13, 19, 37, 45–48) to estimate the social contribution to heterogeneous susceptibility, and the corresponding immunity factor $\lambda_{s}$. The biological contribution, $\lambda_{b}$, is expected to depend on specific details of each infection.

To test this theory, we use empirical data for the COVID-19 epidemic to independently estimate the immunity factor λ. In particular, we apply our previously described epidemic model that features multichannel Bayesian calibration (43) to describe epidemic dynamics in New York City (NYC) and Chicago from the start of the epidemic in mid-March until the end of the first wave around June 15, 2020. This model uses high-quality data on hospitalizations, intensive care unit (ICU) occupancy, and daily deaths to extract the underlying $R_{e}\left(S\right)$ dependence in each of two cities. In addition, we perform a similar analysis of data on individual states in the United States, using data generated by the model in ref. 49. Using both approaches, we find that the locations that were severely impacted by the COVID-19 epidemic show a more pronounced reduction of the effective reproduction number. This effect is much stronger than predicted by classical homogeneous models, suggesting a significant role of heterogeneity. The estimated immunity factor ranges between four and five. Importantly, this represents a transient value of the parameter λ observed on intermediate timescales and dependent on both persistent and short-term heterogeneity. Our estimates of the long-term value of the immunity factor defined by persistent heterogeneity only is considerably lower, about two. This difference explains why achieving the state of TCI after the first wave of the epidemic does not imply long-term herd immunity.

Finally, we integrate the persistent heterogeneity theory into our time-of-infection epidemiological model (43), and project possible outcomes of the second wave of the COVID-19 epidemic during the summer months in NYC and Chicago, using data up to the end of June 2020. By considering the worst-case scenario of a full relaxation of any currently imposed mitigation, we find that the results of the heterogeneity-modified model significantly modify the results from the homogeneous mode. In particular, based on our estimate of the immunity factor, our model predicts no second wave in NYC immediately after release of mitigations in June and up to September 2020, indicating that the TCI has likely been achieved there. Chicago, on the other hand, has not passed the TCI threshold that we infer, but the effects of heterogeneity would still result in a substantial reduction of the magnitude of the second wave there, even under the worst-case scenario. This, in turn, suggests that the second wave can be completely eliminated in such medium-hit locations, if appropriate and economically mild mitigation measures are adopted, including, for example, mask wearing, contact tracing, and targeted limitation of potential superspreading events, through limitations on indoor bars, dining, and other venues. We further investigate the issue of fragility of collective immunity in heterogeneous populations. By allowing rewiring of the social network with time, we demonstrate that the TCI may wane, much like an individual’s acquired immunity may wane due to biological factors. This phenomenon would result in the emergence of secondary epidemic waves after a substantial period of low infection counts.

## Theory of Epidemics in Populations with Persistent Heterogeneity

Following in the footsteps of refs. 10, 19, 22–24, 30, and 32, we consider the spread of an epidemic in a population of individuals who exhibit significant persistent heterogeneity in their susceptibilities to infection α. This heterogeneity may be biological or social in origin, and we assume these factors are independent: $\alpha =\alpha_{b}\alpha_{s}$. Effects of possible correlations between $\alpha_{b}$ and $\alpha_{s}$ have been discussed in ref. 24. The biologically driven heterogeneous susceptibility $\alpha_{b}$ is shaped by variations of several intrinsic factors such as the strength of individuals’ immune responses, age, or genetics. In contrast, the socially driven heterogeneous susceptibility $\alpha_{s}$ is shaped by extrinsic factors, such as differences in individuals’ social interaction patterns (their degree in the network of social interactions). Furthermore, individuals’ different risk perceptions and attitudes toward social distancing may further amplify variations in socially driven susceptibility heterogeneity. We only focus on susceptibility that is a persistent property of an individual. For example, people who have elevated occupational hazards, such as health care workers, typically have higher, steady values of $\alpha_{s}$. Similarly, people with low immune response, highly social individuals (hubs in social networks), or scofflaws would all be characterized by above-average overall susceptibility α.

In this work, we group individuals into subpopulations with similar values of α and describe the heterogeneity of the overall population by the probability density function (pdf) of this parameter, f(α). Since α is a relative measure of individual susceptibilities, without loss of generality, we set $\left\langle \alpha \right\rangle \equiv \int_{0}^{\infty }\alpha f\left(\alpha \right)d\alpha =1$. Each person is also assigned an individual reproduction number $R_{i}$, which is an expected number of people that this person would infect in a fully susceptible population with ⟨α⟩=1. Accordingly, from each subpopulation with susceptibility α, there is a respective mean reproductive number $R_{\alpha }$ to which we refer as infectivity throughout this study. Any correlations between individual susceptibility and infectivity will significantly impact the epidemic dynamics. Such correlations are an integral part of most network-based epidemiological models, due to the assumed reciprocity in underlying social interactions, which leads to $R_{\alpha }\approx \alpha$ (9, 10, 23). In reality, not all transmissions involve face-to-face contacts, and biological susceptibility need not be strongly correlated with infectivity. Therefore, it is reasonable to expect only a partial correlation between α and $R_{\alpha }$.

Let $S_{\alpha }\left(t\right)$ be the fraction of susceptible individuals in the subpopulation with susceptibility α, and let $j_{\alpha }\left(t\right)=-{\dot{S}}_{\alpha }$ be the corresponding daily incidence rate per capita in that subpopulation. At the start of the epidemic, we assume everyone is susceptible to infection: $S_{\alpha }\left(0\right)=1$. The course of the epidemic is described by the following age-of-infection model:

$$-\frac{dS_{\alpha }}{dt}=j_{\alpha }\left(t\right)=\alpha S_{\alpha }\left(t\right)J\left(t\right)$$ [1]

$$J\left(t\right)=\int_{0}^{\infty }\left\langle R_{\alpha }K\left(\tau \right)j_{\alpha }\left(t-\tau \right)\right\rangle d\tau .$$ [2]

Here t is the physical time, and τ is the time since infection for an individual; ⟨…⟩ represents averaging over α with pdf f(α). J(t) is the force of infection, that is, per capita incidence rate in a fully susceptible subpopulation with α=1. $R_{\alpha }$ is the previously introduced infectivity, that is, the mean reproductive number of the subpopulation with susceptibility α. K(τ) is the pdf of the generation interval, which we assume to be independent of α, for the sake of simplicity. The homogeneous version of the age-of-infection model was introduced in the early days of mathematical epidemiology in the classical paper by Kermack and McKendrick (1). It is based on the observation that the force of infection, J(t), is defined by the number of previously infected individuals. The contribution of each individual depends on his/her time since infection τ and is weighted by the infectivity profile K(τ). As shown in ref. 50, the rate of the exponential growth of the epidemic can be inferred from the Laplace transform of K(τ). Well-known compartmentalized models correspond to specific functional forms of K(τ), such as the exponential distribution for the SIR model.

According to Eq. **1**, the fraction of susceptibles in the subpopulation with any given α can be expressed as

$$S_{\alpha }\left(t\right)=\exp\left(-\alpha Z\left(t\right)\right).$$ [3]

Here $Z\left(t\right)\equiv \int_{0}^{t}J\left(t^{\prime }\right)dt^{\prime }$. The total susceptible fraction of the population is related to the moment generating function $M_{\alpha }$ of the distribution f(α) (i.e., the Laplace transform of f(α)) according to

$$S\left(t\right)=\int_{0}^{\infty }f\left(\alpha \right)e^{-\alpha Z\left(t\right)}d\alpha =M_{\alpha }\left(-Z\left(t\right)\right).$$ [4]

Similarly, the effective reproductive number $R_{e}$ can be expressed in terms of the parameter Z,

$$R_{e}\left(t\right)=\frac{1}{\left\langle \alpha \right\rangle }\int_{0}^{\infty }\alpha R_{\alpha }f\left(\alpha \right)e^{-\alpha Z\left(t\right)}d\alpha .$$ [5]

Note that, for Z=0, this expression gives the basic reproduction number $R_{0}=\left\langle \alpha R_{\alpha }\right\rangle /\left\langle \alpha \right\rangle$. This result is reminiscent of the well-known result (23) $R_{0}=\left\langle k_{in}k_{out}\right\rangle /\left\langle k_{in}\right\rangle$ for epidemic spread on a directed network with in and out degrees $k_{in}$ (analogue of our susceptibility α) and $k_{out}$ (analogue of $R_{\alpha }$). Note that, due to our choice of normalization ⟨α⟩=1, the prefactor in Eq. **5** can be omitted. Since both S and $R_{e}$ depend on time only through Z(t), Eqs. **4** and **5** establish a parametric relationship between these two important quantities during the time course of an epidemic. In contrast to the classical case when these two quantities are simply proportional to each other, that is, $R_{e}=SR_{0}$, the relationship in the present theory is nonlinear due to heterogeneity. Eq. **5** was derived by substituting Eq. **1** into Eq. **2**. This leads to the renewal equation for J(t) of the same form as in a homogeneous problem,

$$J\left(t\right)=\int_{0}^{\infty }d\tau K\left(\tau \right)R_{e}\left(t-\tau \right)J\left(t-\tau \right).$$ [6]

Furthermore, by averaging Eq. **1** over all values of α, one arrives at the following heterogeneity-induced modification to the relationship between the force of infection and incidence rate:

$$\frac{dS}{dt}=-S_{e}J.$$ [7]

Here

$$S_{e}\left(t\right)=\int_{0}^{\infty }\alpha f\left(\alpha \right)e^{-\alpha Z\left(t\right)}d\alpha =-\frac{dM_{\alpha }\left(-Z\left(t\right)\right)}{dZ}$$ [8]

is the effective susceptible fraction of the population, which is less than S, due to the disproportionate removal of highly susceptible individuals. Just as with $R_{e}$, it is a nonlinear function of S, defined parametrically by Eqs. **4** and **8**. Further generalization of this theory for the time-modulated age-of-infection model is presented in *SI Appendix*. There, we also discuss the adaptation of this approach for the important special case of a compartmentalized SIR/SEIR model.

One of the striking consequences of the nonlinearity of $R_{e}\left(S\right)$ is that the effective reproduction number could be decreasing at the early stages of an epidemic significantly faster than predicted by homogeneous models. Specifically, for 1−S(t)≃Z(t)≪1, one can linearize the effective reproduction number as

$$R_{e}\approx R_{0}\left(1-\lambda \left(1-S\right)\right).$$ [9]

We named the coefficient λ the *immunity factor* because it quantifies the effect that a gradual buildup of population immunity has on the spread of an epidemic. The classical value of λ is one, but it may be significantly larger in a heterogeneous case. By linearizing Eq. **5** in terms of 1−S≃Z≪1 and dividing the result by $R_{0}=\left\langle \alpha R_{\alpha }\right\rangle$, one gets

$$\lambda =\frac{\left\langle \alpha^{2}R_{\alpha }\right\rangle }{\left\langle \alpha R_{\alpha }\right\rangle }.$$ [10]

As one can see, the value of the immunity factor thus depends both on the statistics of susceptibility α and on its correlation with infectivity $R_{\alpha }$.

We previously defined the overall susceptibility as a combination of biological and social factors: $\alpha =\alpha_{s}\alpha_{b}$. Here $\alpha_{s}$ is a measure of the overall social connectivity or activity of an individual, such as the cumulative time of close contact with other individuals averaged over a sufficiently long time interval (known as node strength in network science). Since the contribution of interpersonal contacts to an epidemic spread is mostly reciprocal, we assume $R_{\alpha }\approx \alpha_{s}$. On the other hand, in our analysis, we neglect a correlation between biological susceptibility and infectivity, as well as between $\alpha_{b}$ and $\alpha_{s}$. Under these approximations, the immunity factor itself is a product of biological and social contributions, $\lambda =\lambda_{b}\lambda_{s}$. Each of them can be expressed in terms of leading moments of $\alpha_{b}$ and $\alpha_{s}$, respectively,

$$\lambda_{b}=\frac{\left\langle \alpha_{b}^{2}\right\rangle }{{\left\langle \alpha_{b}\right\rangle }^{2}}=1+CV_{b}^{2}$$ [11]

$$\lambda_{s}=\frac{\left\langle \alpha_{s}^{3}\right\rangle }{\left\langle \alpha_{s}\right\rangle \left\langle \alpha_{s}^{2}\right\rangle }=1+\frac{CV_{s}^{2}\left(2+\gamma_{s}CV_{s}\right)}{1+CV_{s}^{2}}.$$ [12]

These equations follow from Eq. **10** in the limit $R_{\alpha }=const$ and $R_{\alpha }\approx \alpha$, respectively. Although these equations resemble classical results for $R_{0}$ in heterogeneous networks (8–10, 23), here they describe a completely different effect of suppression of $R_{e}$ in response to depletion of susceptible population S. That is why $\lambda_{s}$ in Eq. **12** is proportional to the third moment of $\alpha_{s}$ instead of the second moment in the case of $R_{0}=\left\langle \alpha R_{\alpha }\right\rangle \approx \alpha_{s}^{2}$. Note that the biological contribution to the immunity factor depends only on the coefficient of variation $CV_{b}$ of $\alpha_{b}$. On the other hand, the social factor $\lambda_{s}$ depends on both the coefficient of variation $CV_{s}$ and the skewness $\gamma_{s}$ of the distribution of $\alpha_{s}$. Due to our normalization, $\left\langle \alpha_{s}\right\rangle \left\langle \alpha_{b}\right\rangle \approx \left\langle \alpha_{s}\alpha_{b}\right\rangle =\left\langle \alpha \right\rangle =1$.

The relative importance of biological and social contributions to the overall heterogeneity of α may be characterized by a single parameter χ. For a log-normal distribution of $\alpha_{b}$, χ appears as a scaling exponent between infectivity and susceptibility: $R_{\alpha }\approx \alpha^{\chi }$ (see *SI Appendix* for details). The corresponding expression for the overall immunity factor is $\lambda =\left\langle \alpha^{2+\chi }\right\rangle /\left\langle \alpha^{1+\chi }\right\rangle$. The limit χ=0 corresponds to a predominantly biological source of heterogeneity, that is, $\lambda \approx \lambda_{b}=1+CV_{\alpha }^{2}$, where $CV_{\alpha }$ is the coefficient of variation for the overall susceptibility. In the opposite limit χ=1, population heterogeneity is primarily of social origin; hence $\lambda \approx \lambda_{s}$ is affected by both $CV_{\alpha }$ and the skewness $\gamma_{\alpha }$ of the pdf f(α). The biological contribution $\lambda_{b}$ depends on specific biological details of the disease and thus is unlikely to be as universal and robust as the social one. For the COVID-19 epidemic, there is no strong evidence of a wide variation in attack rates unrelated to social activity, geographic location, or socioeconomic status. For instance, there is very little age variability in COVID-19 prevalence as reported by the NYC Department of Health (51) based on the serological survey that followed the first wave of the epidemic. Therefore, below, we will largely ignore possible biological heterogeneity, and focus on social heterogeneity.

So far, our discussion has focused on the early stages of epidemics, when the $R_{e}\left(S\right)$ dependence is given by a linearized expression Eq. **9**. To describe the nonlinear regime, we consider a gamma-distributed susceptibility: $f\left(\alpha \right)\approx \alpha^{1/\eta -1}\exp\left(-\alpha /\eta \right)$, where $\eta =CV_{\alpha }^{2}$. In this case, according to Eqs. **4** and **5**, $R_{e}$, $S_{e}$, and S are related by scaling relationships (see *SI Appendix*),

$$S_{e}\left(S\right)=S^{1+\eta }$$ [13]

and

$$R_{e}\left(S\right)=R_{0}S^{\lambda }.$$ [14]

**Fig. 1.**
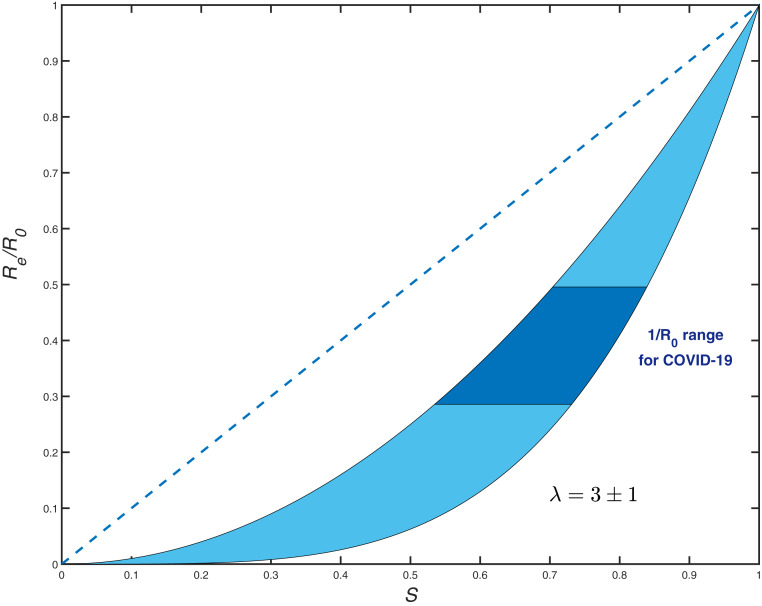
$R_{e}/R$ vs. S dependence for gamma-distributed susceptibility with λ=3±1 (light blue area). The dashed line shows the classical homogeneous result, $R_{e}=R_{0}S$. The dark blue region corresponds to the range $2<R_{0}<3.5$ representative for COVID-19.

The exponent $\lambda =1+\left(1+\chi \right)CV_{\alpha }^{2}=1+\left(1+\chi \right)\eta$ coincides with the early-epidemics immunity factor defined in Eqs. **9** and **10** for a general case. Note that, without correlation (χ=0), both scaling exponents would be the same; this result has been previously obtained for the SIR model in ref. 30 and more recently reproduced in ref. 41 in the context of COVID-19. The scaling behavior $R_{e}\left(S\right)$ is shown in Fig. 1 for λ=3±1. While this range is arbitrary, it includes the empirical values of λ estimated below. This function is dramatically different from the classical linear dependence $R_{e}=SR_{0}$. To emphasize the importance of this difference, Eq. **14** immediately leads to a major revision of the classical result for the HIT $1-S_{0}=1-1/R_{0}$. $S_{0}$ is the fraction of susceptible population at which the growth stops, while $1-S_{0}$ is the relative size of the epidemic at that time. By setting $R_{e}=1$ in Eq. **14**, we obtain

$$1-S_{0}=1-{\left(\frac{1}{R_{0}}\right)}^{1/\lambda }.$$ [15]

Nonlinear modifications to homogeneous epidemiological models similar to Eqs. **13** and **14** have been proposed in the past as plausible descriptions of heterogeneous populations in various contexts. Specifically, they were used as empirical fits to simulations of the SIR model on small-world networks (19), as well as to the behavior of the Agent-Based Model on realistic urban contact networks (18). A conceptual explanation of the origin of a nonlinear relation between S and Re was proposed in refs. 19 and 30. However, the scaling law similar to Eqs. **13** and **14** has not been derived except in a special case of the SIR model without correlation between susceptibility and infectivity (30). As we were finalizing this paper for public release, a preprint by Aguas et al. (52) appeared online that independently obtained our Eqs. **14** and **15** for gamma-distributed susceptibilities. The same result has also been recently obtained in ref. 42. Our approach is more general: It provides the exact mapping of a wide class of heterogeneous well-mixed models onto homogeneous ones, and provides a specific relationship between the underlying statistics of α and $R_{\alpha }$ and the nonlinear functions $R_{e}\left(S\right)$ and $S_{e}\left(S\right)$. Of course, our methodology has the same limitations as the original heterogeneous well-mixed approximation (10). This approximation was shown to provide an adequate description for many classes of networks (19). Additional corrections may still arise, for example, due to clustering and other network structure not captured in its degree (α) distribution.

Our focus on the gamma distribution is well justified by the observation that the social strength $\alpha_{s}$ is approximately exponentially distributed, that is, it is a specific case of the gamma distribution with $\eta =CV_{\alpha }^{2}=1$ (see more discussion of this in the next section). A moderate biological heterogeneity would lead to an increase of the overall $CV_{\alpha }$, but the pdf f(α) will still be close to the gamma distribution family. From the conceptual point of view, it is nevertheless important to understand how the function $R_{e}\left(S\right)$ would change if f(α) had a different functional form. In *SI Appendix*, we present analytic and numerical calculations for two other families of distributions: 1) an exponentially bounded power law $f\left(\alpha \right)\approx e^{-\alpha /\alpha_{+}}/\alpha^{q}$ (q≥1, with an additional cutoff at lower values of α), and 2) the log-normal distribution. In addition, we give an approximate analytic result that generalizes Eq. **14** for an arbitrary skewness of f(α). This generalization works remarkably well for all three families of distributions analyzed in this work. As suggested by Eqs. **11** and **12**, as the distribution becomes more skewed, the range between the χ=0 and χ=1 curves broadens. For instance, for distributions dominated by a power law, $f\left(\alpha \right)\approx 1/\alpha^{q}$, with 3<q<4 and χ=1, λ diverges even though $CV_{\alpha }$ remains finite. This represents a crossover to the regime of so-called scale-free networks (2≤q≤3), which are characterized by zero epidemic threshold yet strongly self-limited dynamics: The epidemic effectively kills itself by immunizing the hubs on the network (10, 23, 53).

## Role of Short-Term Variations in Social Activity

Short-term overdispersion in transmission is commonly presumed to have no effect on the overall epidemic dynamics, aside from the early outbreak often dominated by superspreaders. This would indeed be the case if overdispersed transmission were completely uncorrelated with individual susceptibility. But, since the timescale for an individual’s infectivity (about 2 d) is comparable to a single generation interval (about 5 d) for the COVID-19 epidemics, ignoring such correlations appears unreasonable. We therefore developed a generalization of the theory presented in the previous section, that takes into account a time dependence of individual susceptibilities and infectivities, as well as temporal correlations between them. The theory is presented, using a path integral formulation, in *SI Appendix*. Here we present several important results directly related to the transient suppression of an epidemic and differentiate these effects from herd immunity.

Since fast variations are primarily caused by bursty dynamics of social interactions (54–57), and since heterogeneous biological susceptibility appears subdominant in the context of COVID-19, we set $\alpha_{b}=1$ for all individuals. So α has a purely social origin. Let $a_{i}\left(t\right)=\alpha_{i}+\delta a_{i}\left(t\right)$ be the time-dependent susceptibility of a person, which we associate with a variable level of social activity. Here $\delta a_{i}\left(t\right)$ represents the time-dependent deviation of $a_{i}\left(t\right)$ from its persistent long-term average $\alpha_{i}$. Note that index i labels individuals rather than population groups. The level of social activity quantified by $a_{i}\left(t\right)$ also determines individual infectivity a(t)R around time t. Interestingly, even the classical result for the basic reproduction number in a heterogeneous system, $R_{0}=R\left\langle \alpha^{2}\right\rangle$, needs to be modified due to correlated short-term variations in social activity,

$$R_{0}=R\left(\left\langle \alpha^{2}\right\rangle +\bar{\delta a_{i}^{2}}\right).$$ [16]

Here the bar $\bar{\ldots }$ represents averaging over individual members of the population indexed by i, in contrast with ⟨…⟩, averaging over all subgroups with various values of persistent heterogeneity α.

In the time-dependent generalization of our theory, $R_{e}$ and S no longer have a fixed functional relationship between them. Instead, this relationship becomes nonlocal in time. For instance, our result for the suppression of $R_{e}$ at the early stages of the epidemic is still formally valid, but the effective value of immunity factor λ becomes time dependent, and Eqs. **9** and **10** become

$$\lambda_{e\mathrm{ff}}\left(t\right)=\lambda_{\infty }+\frac{1}{1-S\left(t\right)}\int_{0}^{\infty }\delta \lambda \left(t,t^{\prime }\right)J\left(t-t^{\prime }\right)dt^{\prime }$$ [17]

$$\lambda_{\infty }=\frac{\left\langle \alpha^{3}\right\rangle +\bar{\alpha_{i}\delta a_{i}^{2}}}{\left\langle \alpha^{2}\right\rangle +\bar{\delta a_{i}^{2}}}$$ [18]

$$\delta \lambda \left(t,t^{\prime }\right)=\frac{\bar{\delta a_{i}^{2}\left(t\right)\delta a_{i}\left(t-t^{\prime }\right)}}{\left\langle \alpha^{2}\right\rangle +\bar{\delta a_{i}^{2}}}.$$ [19]

Constant $\lambda_{\infty }$ reflects suppression of $R_{e}$ due to the buildup of the long-term collective immunity. On the other hand, the time-dependent term $\delta \lambda \left(t^{\prime }\right)$ leads to an additional suppression of $R_{e}$ over intermediate timescales. This term has likely played a significant role in shaping the transient self-limiting dynamics during the first wave of COVID-19 epidemic in some hard-hit locations.

Note that, according to Eq. **17**, $\delta \lambda \left(t^{\prime }\right)$ is being averaged with the weight proportional to the force of infection $J\left(t-t^{\prime }\right)$, since $1-S\left(t\right)\approx \int_{0}^{\infty }J\left(y-t^{\prime }\right)dt^{\prime }$. Since $\delta \lambda \left(t,t^{\prime }\right)$ decreases with time difference $t^{\prime }$, its effect on $\lambda_{e\mathrm{ff}}$ should be the strongest during the initial period of fast exponential growth. The initial suppression of the epidemic is caused by the combined effect of mitigation measures and both terms in $\lambda_{e\mathrm{ff}}$. Since $\lambda_{e\mathrm{ff}}>\lambda_{\infty }$, the population may reach the state of TCI earlier than the actual long-term herd immunity determined by persistent heterogeneity. However, this state is fragile and may wane with time. Specifically, as J(t) drops after the first wave, the second term in Eq. **17** gradually decays, bringing $\lambda_{e\mathrm{ff}}\left(t\right)$ closer to $\lambda_{\infty }$. According to Eq. **19**, it is the correlation time of bursty social activity δa(t) that sets the timescale over which this TCI state deteriorates, and the new epidemic wave may get ignited. The relationship between this relaxation time and the duration of a single epidemic wave also determines the typical value of $\lambda_{e\mathrm{ff}}$ during that wave.

Despite a large number of empirical studies of contact networks (54–57), information about the temporal correlations in α(t) or its proxies remains limited. On the other hand, much more is known about parameters of persistent heterogeneity. Recently, real-world networks of face-to-face communications have been studied using a variety of tools, including Radio-frequency identification (RFID) devices (45), Bluetooth and Wi-Fi wearable tags, and smartphone apps (46, 47), as well as census data and personal surveys (13, 37, 48). Despite coming from a wide variety of contexts, the major features of contact networks are remarkably robust. In particular, pdfs of both the degree (the number of contacts per person) and the node strength plotted in log–log coordinates appear nearly constant, followed by a sharp fall after a certain upper cutoff. This behavior is generally consistent with an exponential distribution in $f_{s}\left(\alpha_{s}\right)$ (19, 46, 48), $f\left(\alpha \right)\approx e^{-\alpha /\left\langle \alpha \right\rangle }$. That sets the value of $\eta =CV_{\alpha }^{2}\approx 1$. If not for short-term overdispersion, that would yield $\lambda =\left\langle \alpha_{s}^{3}\right\rangle /\left\langle \alpha_{s}^{2}\right\rangle =3!/2!=3$ according to Eq. **12**. However, with temporal effects taken into account, the buildup of long-term collective immunity is determined by $\lambda_{e\mathrm{ff}}\left(\infty \right)=\lambda_{\infty }$. In order to estimate it, we make a simple model assumption that the short-term overdispersion for a particular individual is proportional to the persistent value of that person’s social activity: $\bar{\delta a_{i}^{2}}\approx \alpha_{i}$. This leads to

$$\lambda_{\infty }=1+\eta \left(1+\chi^{*}\right).$$ [20]

Here $\chi^{*}=\left\langle \alpha^{2}\right\rangle /\bar{a_{i}{\left(t\right)}^{2}}$ is a parameter that measures the relative strength of persistent heterogeneity and the overdispersion on the timescale of a single generation interval. Note that, formally, we recover our original result for λ in the purely persistent case, with $\chi^{*}$ replacing the parameter χ that originally quantified the correlation between infectivity and susceptibility. By assuming the limit of strong short-term overdispersion ($\chi^{*}\ll 1$), we obtain $\lambda_{\infty }\approx 2$. This estimate is consistent with numerical simulations of the agent-based epidemiological model on urban contact networks carried out in ref. 18.

**Fig. 2.**
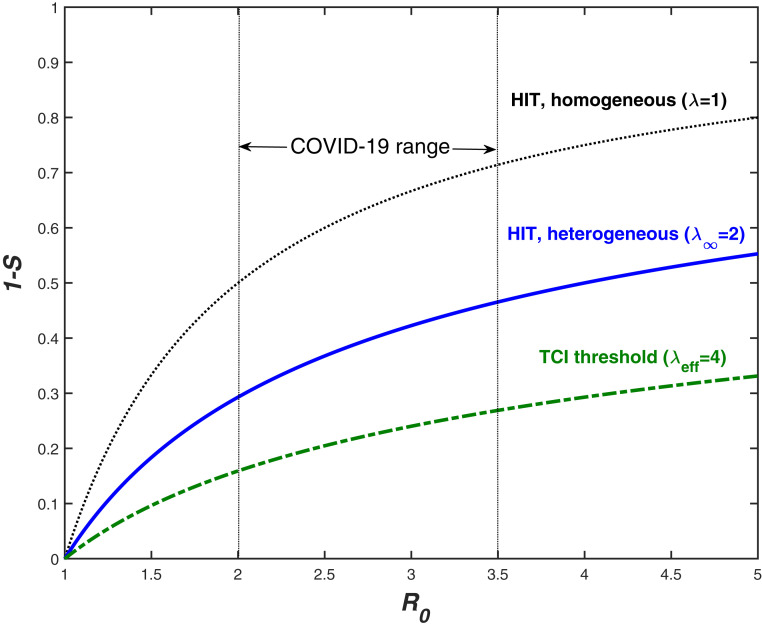
TCI threshold (dot-dashed), long-term heterogeneous HIT (solid), and homogeneous HIT (dotted) for various values of $R_{0}$ (*x* axis). HIT (solid line) is determined by persistent heterogeneity. The corresponding immunity factor $\lambda_{\infty }\approx 2$ was estimated from Eq. **20** assuming strong short-term overdispersion ($\chi^{*}\ll 1$) and the exponential distribution of α (η=1). For transient behavior, $\lambda_{e\mathrm{ff}}\approx 4$ is assumed based on analysis of empirical data for COVID-19 epidemic in select locations.

As shown in *SI Appendix*, the very same value of $\lambda_{\infty }$ should be used as a scaling exponent for long-term behavior of $R_{e}\left(S\right)$. Therefore, HIT is set by Eq. **15** with $\lambda =\lambda_{\infty }\approx 2$. Its value is plotted vs. $R_{0}$ in Fig. 2, along with the homogeneous result, and the estimated threshold of TCI. To estimate the corresponding transient immunity factor $\lambda_{e\mathrm{ff}}$, we analyze the empirical data for the first wave of the COVID-19 epidemic, below.

## Application to the COVID-19 Epidemic

**Fig. 3.**
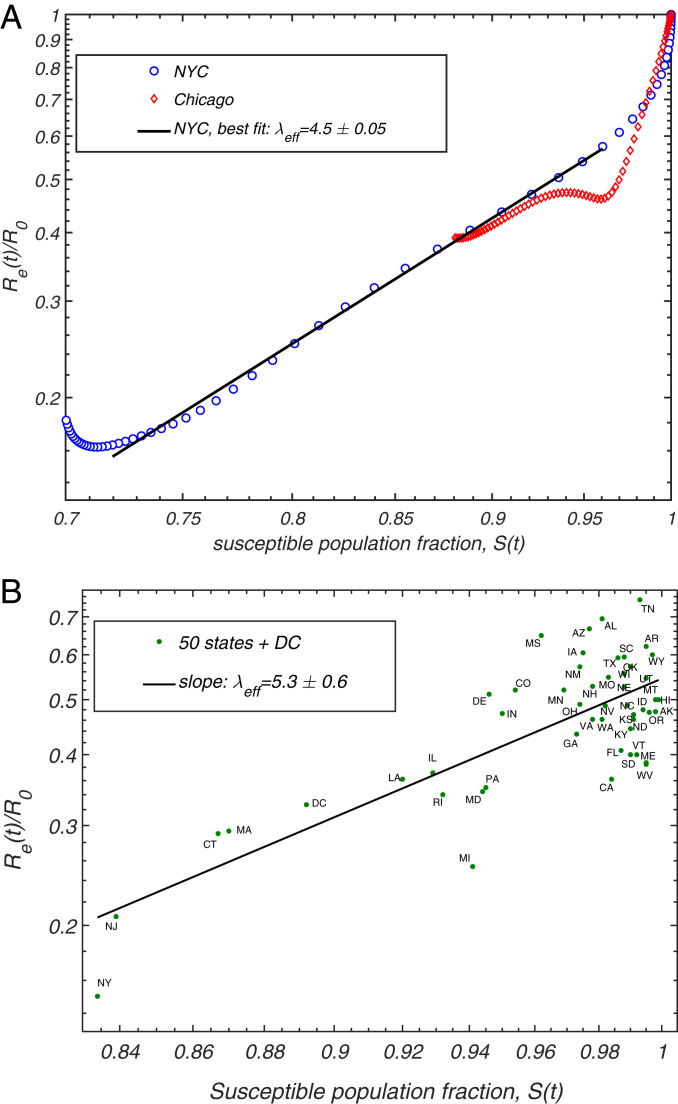
Correlation between the relative reduction in the effective reproduction number $R_{e}\left(t\right)/R_{0}$ (*y* axis) with the susceptible population S(t). (*A*) The progression of these two quantities for NYC and Chicago, as given by the epidemiological model described in ref. 43. (*B*) The scatter plot of $R_{e}\left(t_{0}\right)/R_{0}$ and $S\left(t_{0}\right)$ in individual states of the United States, evaluated in ref. 49 ($t_{0}$ is the latest date covered in that study).

The COVID-19 epidemic reached the United States in early 2020, and, by March, it was rapidly spreading across multiple states. The early dynamics was characterized by a rapid rise in the number of cases, with doubling times as low as 2 d. In response to this, the majority of states imposed a broad range of mitigation measures including school closures, limits on public gatherings, and stay-at-home orders. In many regions, especially the hardest-hit ones like NYC, people started to practice some degree of social distancing even before government-mandated mitigation. In order to quantify the effects of heterogeneity on the spread of the COVID-19 epidemic, we apply the Bayesian age-of-infection model described in ref. 43 to NYC and Chicago (see *SI Appendix* for details). For both cities, we have access to reliable time series data on hospitalization, ICU room occupancy, and confirmed daily deaths due to COVID-19 (51, 58–60). We used these data to perform multichannel calibration of our model (43), which allows us to infer the underlying time progression of both S(t) and $R_{e}\left(t\right)$. The fits for $R_{e}\left(S\right)$ for both cities are shown in Fig. 3*A*. In both cases, a sharp drop of $R_{e}$ that occurred during the early stage of the epidemic is followed by a more gradual decline. For NYC, there is an extended range over which $R_{e}\left(S\right)$ has a constant slope in logarithmic coordinate. This is consistent with the power law behavior predicted by Eq. **14**, with the slope corresponding to transient immunity factor $\lambda_{e\mathrm{ff}}=4.5\pm 0.05$. Chicago exhibits a similar behavior but over a substantially narrower range of S. This reflects the fact that NYC was much harder hit by the COVID-19 epidemic. Importantly, the range of dates we used to estimate the immunity factor corresponds to the time interval after state-mandated stay-at-home orders were imposed, and before the mitigation measures began to be gradually relaxed. The signatures of the onset of the mitigation and of its partial relaxation are clearly visible on both ends of the constant-slope regime. To examine the possible effects of variable levels of mitigation on our estimates of $\lambda_{e\mathrm{ff}}$, in *SI Appendix*, we repeated our analysis in which $R_{e}\left(t\right)$ was corrected by Google’s community mobility report in these two cities (61) (see *SI Appendix*). Although the range of data consistent with the constant slope shrank somewhat, our main conclusion remains unchanged. This provided us with a lower-bound estimate for the transient immunity factor: $\lambda_{e\mathrm{ff}}=4.1\pm 0.1$.

To test the sensitivity of our results to details of the epidemiological model and choice of the region, we performed an alternative analysis based on the data reported in ref. 49. In that study, the COVID-19 epidemic was modeled in each of the 50 US states and the District of Columbia. Because of the differences in population density, level of urbanization, use of public transport, etc., different states were characterized by substantially different initial growth rates of the epidemic, as quantified by the basic reproduction number $R_{0}$. Furthermore, the time of arrival of the epidemic also varied a great deal between individual states, with states hosting major airline transportation hubs being among the earliest ones hit by the virus. As a result of these differences, at any given time, the infected fraction of the population differed significantly across the United States (49). We use state level estimates of $R_{e}\left(t\right)$, $R_{0}$, and S(t) as reported in ref. 49 to construct the scatter plot $R_{e}\left(t_{0}\right)/R_{0}$ vs. $S\left(t_{0}\right)$ shown in Fig. 3*B*, with $t_{0}$ chosen to be the last reported date in that study, May 17, 2020. By performing the linear regression on these data in logarithmic coordinates, we obtain the fit for the slope $\lambda_{e\mathrm{ff}}=5.3\pm 0.6$ and for S=1 intercept around 0.54. In *SI Appendix*, Fig. S3, we present an extended version of this analysis for the 10 hardest-hit states and the District of Columbia, which takes into account the overall time progression of $R_{e}\left(t\right)$ and S(t), and gives similar estimate $\lambda_{e\mathrm{ff}}=4.7\pm 1.5$. Both estimates of the immunity factor based on the state data are consistent with our earlier analysis of NYC and Chicago. In light of our theoretical picture, this value of this transient immunity factor, $\lambda_{e\mathrm{ff}}≃4$, is set by the pace of the first epidemic wave in the United States. As expected, it exceeds our estimate of $\lambda_{\infty }\approx 2$ associated with persistent heterogeneity and responsible for the long-term herd immunity.

**Fig. 4.**
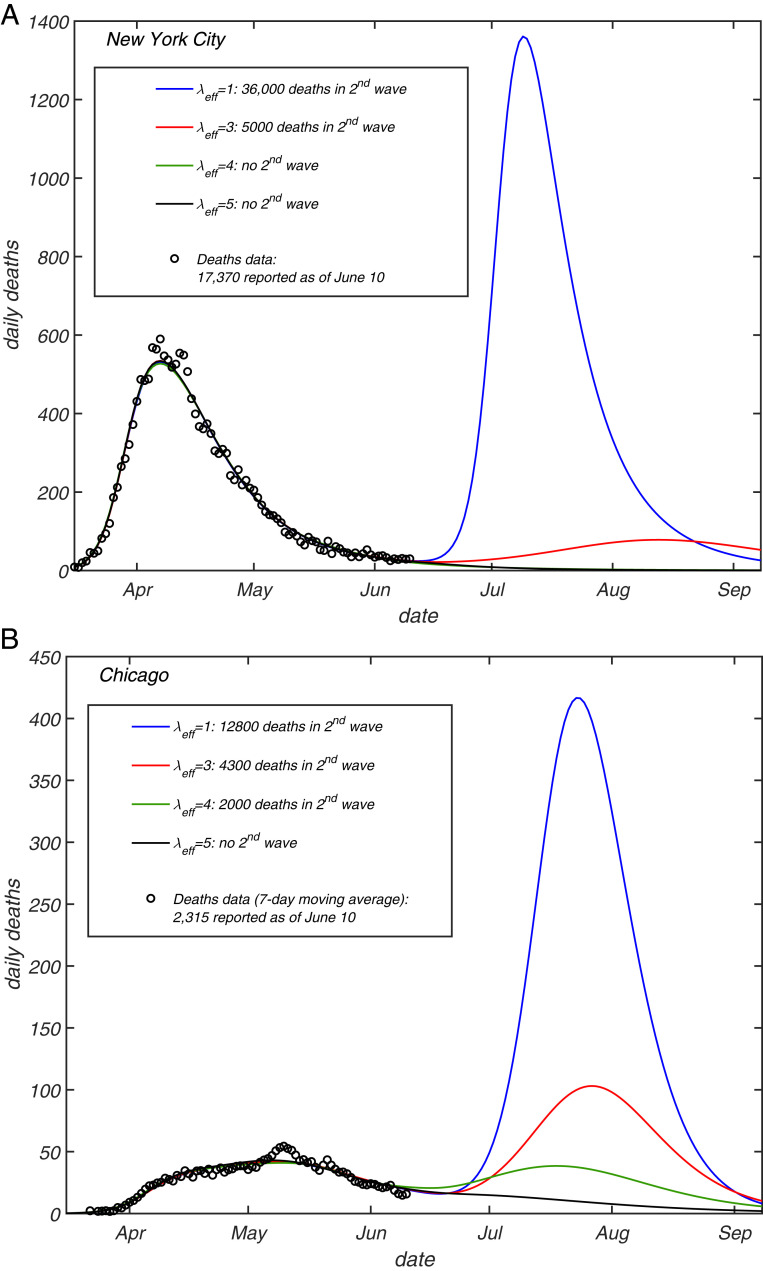
Projections of daily deaths under the hypothetical scenario in which any mitigation is completely eliminated as of June 15, 2020, for (*A*) NYC and (*B*) Chicago. Different curves correspond to different values of the transient immunity factor $\lambda_{e\mathrm{ff}}=1$ (blue), 3 (red), 4 (green), and 5 (black lines). The model described in ref. 43 was fully calibrated on daily deaths (circles), ICU occupancy, and hospitalization data up to the end of May. See *SI Appendix* for additional details, including CIs.

We can now incorporate this transient level of heterogeneity into our epidemiological model, and examine how future projections change as a result of this modification. This is done by plugging scaling relationships given by Eqs. **13** and **14** into the force of infection and incidence rate equations of the original model. These equations are similar to Eqs. **6** and **7**, but also include time modulation due to the mitigation and a possible seasonal forcing (see *SI Appendix* for more details). After calibrating the model by using the data streams on ICU occupancy, hospitalization, and daily deaths up to the end of May, we explore a hypothetical worst-case scenario in which any mitigation is completely relaxed as of June 15, in both Chicago and NYC. In other words, the basic reproduction number $R_{0}$ is set back to its value at the initial stage of the epidemic, and the only factor limiting the second wave is the partial or full TCI, $R_{e}=R_{0}S^{\lambda }$. The projected daily deaths for each of the two cities under this (unrealistically harsh) scenario are presented in Fig. 4 for various values of λ. For both cities, the homogeneous model (λ=1, blue lines) predicts a second wave which is larger than the first one, with an additional death toll of around 35,000 in NYC and 12,800 in Chicago. The magnitude of the second wave is greatly reduced by heterogeneity, resulting in no second wave in either of the two cities for λ=5 (black lines). Even for a modest value λ=3 (red lines), which is less than our estimate, the second wave is dramatically reduced in both NYC and Chicago (by about 90% and 70%, respectively).

Note that our predictions about the second wave in NYC and Chicago have been made based on the data up to June 10, 2020 and extended up to early September 2020. The real epidemic dynamic in both cities during this time interval was consistent with the “no second wave” scenario shown in Fig. 4. However, one must be warned against using it to put form bounds on $\lambda_{e\mathrm{ff}}$, since we considered the worst-case scenario of full release of mitigation to prepandemic levels. In reality, some mitigation measures, for example, mask wearing and social distancing, stayed in place. Ultimately, second waves broke out in both cities in the late fall. The mechanisms leading to gradual degradation of the TCI state are described in the next section.

## Fragility of TCI

One of the consequences of the bursty nature of social interactions is that the state of TCI gradually wanes due to changes of individual social interaction patterns on timescales longer than a single generation interval. This may be viewed as a slow rewiring of social networks. In the context of the COVID-19 epidemic, individual responses to mitigation factors such as stay-at-home orders may differ across the population. When mitigation measures are relaxed, individual social susceptibility $\alpha_{s}$ inevitably changes. The impact of these changes on collective immunity depends on whether each person’s $\alpha_{s}$ during and after the mitigation are sufficiently correlated. For example, the TCI state would be compromised if people who practiced strict self-isolation compensated for it by an above-average social activity after the first wave of the epidemic has passed.

To illustrate the effects of postmitigation rewiring of social networks, we consider a simple modification of the heterogeneous model with no persistent heterogeneity (α=1 for everyone) and exponentially distributed instantaneous levels of social activity $a_{i}\left(t\right)$. This corresponds to $\lambda_{e\mathrm{ff}}\left(0\right)=3$ and $\lambda_{e\mathrm{ff}}\left(\infty \right)=\lambda_{\infty }=1$. In this model, each individual completely changes the set of his/her social connections at some timescale $\tau_{s}$. These changes destroy heterogeneity, giving rise to gradual relaxation of susceptible fraction $S_{a}$ toward its overall mean value S. To model this, we modify Eq. **1** to include a simple relaxation term,

$${\dot{S}}_{a}=-\alpha S_{a}J-\frac{1}{\tau_{s}}\left(S_{a}-S\right).$$ [21]

Epidemiological models with rewiring of underlying social networks have been studied before (62) (see ref. 63 for a review), but under a constraint that the individual level of social activity quantified by network degree is preserved. In contrast, the dynamics described above stems from the individual level of social activity $\alpha_{s}$ changing in time.

**Fig. 5.**
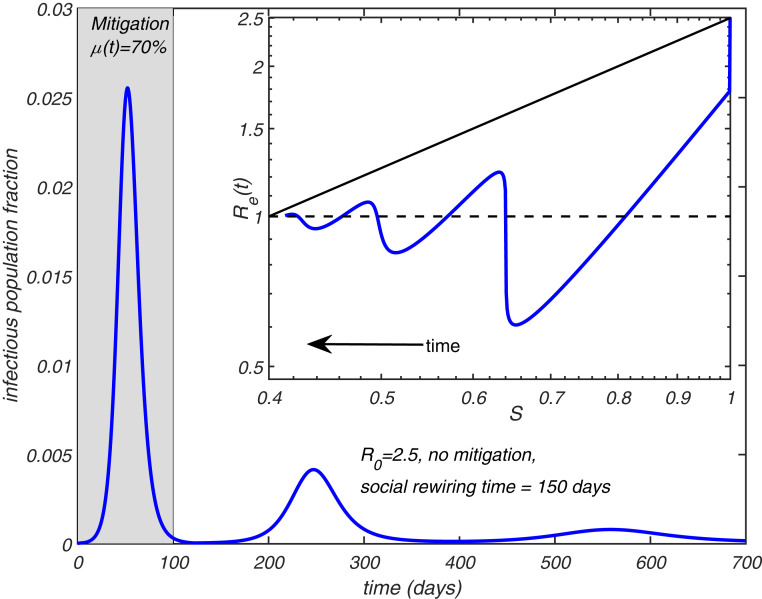
Effect of social rewiring on the epidemic dynamics. The time course of an epidemic in a heterogeneous SIR model with $R_{0}=2.5$ and λ=3. During the first 100 d, a mitigation factor μ=0.7 is applied. Social networks gradually rewire with a time constant $\tau_{s}=150$ d. The figure shows multiple waves. (*Inset*) $R_{e}\left(t\right)$ plotted as a function of S(t). Solid black line shows the homogeneous limit reached after multiple waves.

We simulate the full heterogeneous SIR model (see *SI Appendix* for details) in which Eq. **1** was replaced with Eq. **21**. Fig. 5 shows the results of this simulation, where the first wave of the epidemic is mitigated, thereby reducing the effective reproduction number $R_{0}=2.5$. During the course of the mitigation, $R_{0}$ is multiplied by μ=0.7. After the mitigation measures have been lifted at the end of the first wave, the population is positioned slightly below the TCI threshold, preventing the immediate start of the second wave. However, gradual rewiring of the social network with time constant $\tau_{s}=150$ d ultimately results in the second and even the third wave of the epidemic (Fig. 5). Fig. 5, *Inset* shows $R_{e}\left(t\right)$ plotted as a function of S(t) in this epidemic. Note that each of the waves follows the power law relationship between $R_{e}\left(t\right)\approx S{\left(t\right)}^{\lambda }$ predicted by Eq. **14**. Since constant rewiring eliminates correlations in individual social activity on scales longer than $\tau_{s}$, the epidemic stops after multiple waves bring the total fraction of infected individuals close to the unmodified (homogeneous) HIT $1/R_{0}$. Note, however, that, in this case, there is almost no overshoot, and thus the final size of the epidemic is reduced compared to the case of a purely homogeneous and unmitigated epidemic.

## Discussion

In this work, we have demonstrated how the interplay between short-term overdispersion and persistent heterogeneity in a population leads to dramatic changes in epidemic dynamics on multiple timescales, transient suppression of the epidemic during its early waves all the way up to the state of long-term herd immunity. First, we developed a general approach that allows for the persistent heterogeneity to be easily integrated into a wide class of traditional epidemiological models in the form of two nonlinear functions $R_{e}\left(S\right)$ and $S_{e}\left(S\right)$, both of which are fully determined by the statistics of individual susceptibilities and infectivities. Furthermore, $R_{e}\left(S\right)$ is largely defined by a single parameter, the immunity factor λ, introduced in our study. Like susceptibility itself, λ has two contributions: biological and social (Eqs. **11** and **12**).

We then expanded our approach to include effects of time dependence of individual social activity, and, in particular, of likely correlations over the timescale of a single generation interval. While our results for purely persistent heterogeneity confirmed and corroborated that HIT would be suppressed compared to the homogeneous case, addition of temporal variations led to a dramatic revision of that simple narrative. Both persistent heterogeneity and short-term overdispersion contributions lead first to a slowdown of a fast-paced epidemic, and to its medium-term stabilization. However, this state of TCI is fragile and does not constitute long-term herd immunity. HIT is indeed suppressed, but only due to the persistent heterogeneity. This suppression is significantly weaker than the initial stabilization responsible for the TCI state reached after the first wave of a fast-paced epidemic.

Among other implications of the TCI phenomenon is the suppression of the so-called overshoot. Namely, it is well known that most models predict that an epidemic will not stop once HIT is passed, ultimately reaching a significantly larger cumulative attack rate, FSE. Multiple prior studies (9, 10, 20, 21, 30, 34) have shown that FSE would be suppressed by persistent heterogeneity, similarly to HIT. In *SI Appendix*, we present a simple result that unifies several previously studied limiting cases, and gives an explicit equation for the FSE for the gamma-distributed susceptibility and variable level of its correlation with infectivity. However, because of the transient suppression of the early waves of the epidemic discussed in this work, the overshoot effect would be much weaker or essentially eliminated. For instance, our simple rewiring model demonstrates how the epidemic, after several waves, ultimately reaches HIT level, but does not progress much beyond it (Fig. 5). The FSE result may still be used, but primarily as an estimate for the size of the first wave of an (unmitigated) epidemic. In that case, the transient value of immunity factor $\lambda_{e\mathrm{ff}}$ should be assumed.

By applying our theory to the COVID-19 epidemic, we found evidence that the hardest-hit areas, such as NYC, have likely passed TCI threshold by the end of the first wave, but are less likely to have achieved real long-term herd immunity. Other places that had intermediate exposure, such as, for example, Chicago, while still below the TCI threshold, have their effective reproduction number reduced by a significantly larger factor than predicted by traditional epidemiological models. This gives a better chance of suppressing the future waves of the epidemic in these locations by less disruptive measures than those used during the first wave, for example, by using masks, social distancing, contact tracing, control of potential superspreading events, etc. However, similar to the case of NYC, transient stabilization of the COVID-19 epidemic in Chicago will eventually wane. As for the permanent HIT, although suppressed compared to classical value, it definitely has not yet been passed in those two locations.

In a recent study (35), the reduction of HIT due to heterogeneity has been illustrated using a toy model. In that model, 25% of the population was assumed to have their social activity reduced by 50% compared to a baseline, while another 25% had their social activity elevated twofold. The rest of the population was assigned the baseline level of activity. According to Eq. **12**, the immunity factor in that model is λ=1.54. For this immunity factor, Eq. **15** predicts HIT at $S_{0}=64\%$, 55%, and 49%, for $R_{0}=2$, 2.5, and 3, respectively. Despite the fact that the model distribution is not gamma shaped, these values are in a very good agreement with the numerical results reported in ref. 35: $S_{0}=62.5\%$, 53.5%, and 47.5%, respectively.

Thus there is a crucial distinction between the persistent heterogeneity, short-term variations correlated over the timescale of a single generation interval, and overdispersion in transmission statistics associated with short-term superspreading events (12, 13, 16, 26–28). In our theory, a personal decision to attend a large party or a meeting would only contribute to persistent heterogeneity if it represents a recurring behavioral pattern. On the other hand, superspreading events are shaped by short-time variations in individual infectivity (e.g., a person during the highly infectious phase of the disease attending a large gathering). Hence, the level of heterogeneity inferred from the analysis of such events (12, 27) would be significantly exaggerated and should not be used to estimate the TCI threshold and HIT. Specifically, the statistics of superspreading events is commonly described by the negative binomial distribution with dispersion parameter k estimated to be in the range 0.1 to 0.3 for COVID-19 (28, 64, 65). This is much stronger overdispersion than the value k=1 estimated from persistent heterogeneity, based on the exponential distribution of α. Thus, persistent heterogeneity is a weaker source of variation compared to short-term variations. According to ref. 12, this is consistent with the expected value of the individual-level reproduction number $R_{i}$ drawn from a gamma distribution with the shape parameter k≃0.1…0.3. This distribution has a very high coefficient of variation, $CV^{2}=1/k≃3\ldots 10$. In the case of a perfect correlation between individual infectivity and susceptibility α, this would result in an unrealistically high estimate of the immunity factor: $\lambda =1+2CV^{2}=1+2/k≃7\ldots 20$. For this reason, according to our perspective and calculation, the final size of the COVID-19 epidemic may have been substantially underestimated in ref. 31. Similarly, the degree of heterogeneity assumed in other recent studies (32, 52) is considerably larger than our estimates. Based on our analysis, the value of the immunity factor λ depends on the pace of the epidemic and on the timescale under consideration. We estimated its long-term value (responsible for the permanent HIT) as $\lambda_{\infty }\approx 2$. However, the transient values are expected to be higher, especially during the first several waves of COVID-19 in select locations, characterized by large growth rates. Our analysis of the empirical data in NYC and Chicago indicates that the slowdown of the epidemic dynamics in those locations was consistent with λ≈4. In Table 1, we present our estimates of the factor by which $R_{e}$ is transiently suppressed as a result of depletion of susceptible population in selected locations in the world, as of early June 2020, as well as the predicted long-term suppression related to acquisition of a partial herd immunity.

**Table 1.** Effects of heterogeneity on suppression of the effective reproduction number $R_{e}$ in selected locations

		Attack rate, 1−S, %		$R_{e}$ suppression	
Location	Deaths, per 1,000 (refs.)	Estimated	Seroprevalence (refs.)	Transient	Long-term
NYC	2.1 (51)	30	23 (68)	0.24 to 0.33	0.50 to 0.58
Lombardy	1.7 (69)	24	23 (70, 71)	0.33	0.58
London	0.9 (72)	13	13 (73)	0.58	0.75
Chicago	0.9 (58)	13	20 (74)	0.41 to 0.58	0.64 to 0.75
Stockholm	0.9 (75, 76)	13	12 (76)	0.58	0.75

The transient and long-term suppression coefficients $R_{e}/R_{0}$ are calculated using $\lambda_{e\mathrm{ff}}=4$ and $\lambda_{\infty }=2$ respectively. Fraction of susceptible population Sas of early June 2020 is estimated from the cumulative reported death count per capita, assuming the infection fatality rate of 0.7% (67). This estimate is supplemented by seroprevalence data for late spring–early summer 2020.

Finally, we summarize the assumptions and limitations of our study. First, we assume a long-lasting biological immunity of recovered individuals. Second, our approach is based on the well-mixed approximation in which geographic heterogeneity as well as nontrivial properties of the contact network (clustering, degree–degree correlations, etc.) are ignored. In addition, our description of transient epidemic dynamics is based on the approximation of a constant $\lambda_{e\mathrm{ff}}$, while gradual degradation of TCI with time is illustrated using a simplified model, Eq. **21**. A generalization of the present model including explicit description of stochastic social activity is needed (see ref. 66). Furthermore, additional calibration based on long-term empirical data is required before our approach can be used for reliably guiding policy decisions during an epidemic.

Population heterogeneity manifests itself at multiple scales. At the most coarse-grained level, individual cities or even countries can be assigned some level of susceptibility and infectivity, which inevitably vary from one location to another, reflecting differences in population density and its connectivity to other regions. Such spatial heterogeneity will result in self-limiting epidemic dynamics at the global scale. For instance, hard-hit hubs of the global transportation network, such as NYC during the COVID-19 epidemic, would gain full or partial TCI, thereby limiting the spread of infection to other regions during future waves of the epidemic. This might be a general mechanism that ultimately limits the scale of many pandemics, from the Black Death to the 1918 influenza.

## Supplementary Material

Supplementary File

## Data Availability

All study data are included in the article and *SI Appendix*.
