# Supplementary material for: Time-dependent heterogeneity leads to transient suppression of the COVID-19 epidemic, not herd immunity
Source: Proc Natl Acad Sci U S A. 2021 Apr 8;118(17):e2015972118. doi: 10.1073/pnas.2015972118 (PMC8092384; doi:10.1073/pnas.2015972118)
Supplement: Supplementary File [file pnas.2015972118.sapp.pdf]

PNAS
www.pnas.org

Supplementary Information for

Time-dependent heterogeneity leads to transient suppression of the COVID-19 epidemic, not herd immunity

Alexei V. Tkachenko, Sergei Maslov, Ahmed Elbanna, George N. Wong, Zachary J. Weiner and Nigel Goldenfeld

Alexei V. Tkachenko
E-Mail: oleksiyt@bnl.gov
Sergei Maslov
E-mail: maslov@illinois.edu

This PDF file includes:

Supplementary text
Figs. S1 to S6 (not allowed for Brief Reports)
SI References

Supporting Information Text

Derivation of quasi-homogeneous model.

Age-of-infection model. We start with the same age-of-infection model as described in the main text, but include additional time-dependent modulation of the force of infection :

$$J(t) = \mu(t) \left\langle \int_0^\infty d\tau R_\alpha K(\tau) j_\alpha(t-\tau) \right\rangle \quad \text{[S1]}$$

Here, the modulation factor $\mu(t)$ can be due (e.g.) to mitigation measures or seasonal forcing. Due to this modification, Eq. (5) should be rewritten as follows:

$$\frac{R_e(t)}{\mu(t)R_0} \equiv S_R(t) = \frac{1}{R_0} \int_0^\infty \alpha R_\alpha f(\alpha) e^{-\alpha Z(t)} d\alpha \quad \text{[S2]}$$

Here $R_0 = \int_0^\infty \alpha R_\alpha f(\alpha) d\alpha$ is the basic reproduction number. As a reminder, we set $\langle \alpha \rangle = 1$. Now one can write the renewal equation for force of infection which is formally identical to the one for a homogeneous case:

$$J(t) = \mu(t) R_0 \int_0^\infty d\tau K(\tau) S_R(t-\tau) J(t-\tau) \quad \text{[S3]}$$

As discussed in the main text, equations for incidence rate $\dot{S}$, S_e and S complete our quasi-homogeneous description:

$$\frac{dS(t)}{dt} = -S_e(t) J(t) \quad \text{[S4]}$$

$$S = \int_0^\infty f(\alpha) e^{-\alpha Z(t)} d\alpha \quad \text{[S5]}$$

$$S_e = \int_0^\infty \alpha f(\alpha) e^{-\alpha Z(t)} d\alpha = -\frac{\partial S(Z)}{\partial Z} \quad \text{[S6]}$$

The set of Eqs.(S3)-(S4) completely describes the epidemic dynamics, as long S_R and S_e are specified as functions of fraction of susceptible population, S.

Compartmentalized SIR/SEIR models. The basic SIR and SIER models can be viewed as particular cases of the age-of infection model discussed above. However, because of their great importance and wide use, we present our construction for a specific case of SEIR:

$$\dot{S}_\alpha = -\alpha S_\alpha J \quad \text{[S7]}$$

$$\dot{E}_\alpha = \alpha S_\alpha J - \gamma_E E_\alpha \quad \text{[S8]}$$

$$\dot{I}_\alpha = \gamma_E E_\alpha - \gamma_I I_\alpha \quad \text{[S9]}$$

Here, $J(t) = \mu(t)\gamma_I \int_0^\infty R_\alpha I_\alpha f(\alpha) d\alpha$ is force of infection. We define infectivity-weighted "Exposed" and "Infectious" fractions as

$$E(t) = \frac{1}{R_0} \int_0^\infty R_\alpha E_\alpha f(\alpha) d\alpha \quad \text{[S10]}$$

$$I(t) = \frac{J}{\gamma_I \mu(t) R_0} = \frac{1}{R_0} \int_0^\infty R_\alpha I_\alpha f(\alpha) d\alpha \quad \text{[S11]}$$

This leads to a complete description of epidemic dynamics with three ordinary differential equations, formally equivalent to those for the homogeneous case:

$$\dot{S}(t) = J(t) \int_0^\infty \alpha S_\alpha f(\alpha) d\alpha = -\gamma_I R_0 \mu(t) S_e(t) I(t) \quad \text{[S12]}$$

$$\dot{E}(t) = \frac{J}{R_0} \int_0^\infty \alpha R_\alpha S_\alpha d\alpha - \gamma_E E(t) = \gamma_I R_0 \mu(t) S_R(t) I(t) - \gamma_E E(t) \quad \text{[S13]}$$

$$\dot{I}(t) = \gamma_E E(t) - \gamma_I I(t) \quad \text{[S14]}$$

Eqs.(S2),S5 and (S6) relating functions S_R, S and S_e can be recovered by using exponential Anzats, $S_\alpha = e^{-\alpha Z}$.

Correlation parameter and scaling relationship between infectivity and susceptibility. Below we consider a model in which biological susceptibility α_b is correlated neither with infectivity nor with social strength α_s of an individual. On the other hand, both the overall susceptibility and infectivity are proportional to α_s. Let f_x and f_y be probability density functions (pdfs) of variables $x \equiv \ln \alpha_s$ and $y \equiv \ln \alpha_b$. It is reasonable to assume a log-normal distribution for α_b, since biological susceptibility can be modeled as a product of several random factors (due to age, gender, genetics, pre-existent conditions, etc). This corresponds to a Gaussian form for f_y with variance σ^2 and mean $-\sigma^2/2$ (assuming normalization $\langle\alpha_b\rangle = 1$). For a given value of α, this translates into Gaussian distribution of variable x with the same variance, and mean $\ln\alpha + \sigma^2/2$. This allows us to calculate the average α_s which is proportional to R_α:

$$R_\alpha \sim \langle\alpha_s\rangle \sim \frac{\int f_x(x)\exp\left(x - \frac{(x-\ln\alpha-\sigma^2/2)^2}{2\sigma^2}\right)dx}{\int f_x(x)\exp\left(-\frac{(x-\ln\alpha-\sigma^2/2)^2}{2\sigma^2}\right)dx} \quad \text{[S15]}$$

This integral can be evaluated by the method of steepest descents: for most pdfs f_x and f_y, will be dominated by the vicinity of point x_0 defined by the condition $f'(x_0)/f(x_0) = (x_0/\sigma^2 - 1/2)$. By expanding $\ln f(x)$ in $x' = x - x_0$, we obtain $f_x(x') \approx f(x_\sigma)\exp(rx' - \kappa x'^2/2)$, where $r = f'(x_0)/f(x_0) = x_0/\sigma^2 - 1/2$ and $\kappa = -f''(x_0)/f(x_0) + r^2$. After substituting this Gaussian approximation for f_x back into the above equation, we obtain the scaling relationship between α and R_α

$$R_\alpha \sim \exp\left(\frac{(\sigma^2+\ln\alpha)^2 - (\ln\alpha)^2}{2\sigma^2(1+\kappa\sigma^2)}\right) \sim \alpha^\chi \quad \text{[S16]}$$

Here $\chi = 1/(1+\kappa\sigma^2)$.

Functions $S_R(S)$ and $S_e(S)$. According to Eq.(S5), function $S(Z)$ is directly related to the moment generating function M_α for pdf $f(\alpha)$

$$S = \langle e^{-\alpha Z}\rangle_\alpha = M_\alpha(-Z) = 1 - Z + \frac{\langle\alpha^2\rangle Z^2}{2} - \frac{\langle\alpha^3\rangle Z^3}{6} + \cdots \quad \text{[S17]}$$

This function also determines the effective fraction of susceptible population S_e, Eq.(S6):

$$S_e = \langle \alpha e^{-\alpha Z}\rangle_\alpha = -\frac{dS}{dZ} \quad \text{[S18]}$$

Once effective susceptible fraction S_e is expressed as function of S, it completely determines how S_R (and hence R_e) behaves in both limiting cases of strong and weak correlations, respectively:

$$S_R^{(\chi)} = \begin{cases} \langle\alpha e^{-\alpha Z}\rangle_\alpha = -dS/dZ = S_e, & \chi = 0 \\ \frac{1}{\langle\alpha^2\rangle}\frac{dS^2}{dZ^2} = \frac{S_e}{\langle\alpha^2\rangle}\frac{dS_e}{dS}, & \chi = 1 \end{cases} \quad \text{[S19]}$$

Application to specific distributions of susceptibility.

Gamma distribution. Consider the gamma distribution with $\langle\alpha\rangle = 1$ and $CV_\alpha^2 = \eta$:

$$f(\alpha) \sim \alpha^{1/\eta-1}\exp(-\alpha/\eta) \quad \text{[S20]}$$

By using Eqs.(S2),(S5),(S6), we obtain:

$$S = (1+\eta Z)^{-1/\eta} \quad \text{[S21]}$$

$$S_e = (1+\eta Z)^{-1/\eta-1} = S^{1+\eta} \quad \text{[S22]}$$

$$S_R = (1+\eta Z)^{-(1+(\chi+1)/\eta)} = S^\lambda \quad \text{[S23]}$$

This leads to the scaling relationship $R_e = R_0 S^\lambda$, Eq. (14).

Truncated power law distribution. We now consider power law distributed α, $f(\alpha) \sim 1/\alpha^{1+s}$ $(s > 0)$, with upper and lower cut-offs, $\epsilon\alpha_+$ and α_+, respectively. If the upper cut-off is implemented as an exponential factor $\exp(-\alpha/\alpha_+)$, we recover the functional form identical to the gamma distribution, Eq. (S20) discussed above, but with negative values of the shape factor:

$$f(\alpha) = \frac{\alpha_+^{q-1}\exp(-\alpha/\alpha_+)}{\alpha^q\Gamma(1-q,\epsilon)} \quad \text{[S24]}$$

Due to the normalization $\langle\alpha\rangle = 1$,

$$\alpha_+ = \frac{\Gamma(1-q,\epsilon)}{\Gamma(2-q,\epsilon)}. \quad \text{[S25]}$$

In the case of gamma distribution, the coefficient of variation CV_α would completely determine the overall shape of pdf. For power law with exponent $1 \leq q \leq 3$, the value of $\eta = CV^2$ sets the dynamic range the between upper and lower cut-offs, i.e. the parameter ϵ:

$$1 + \eta = \langle \alpha^2 \rangle = \frac{\Gamma(1-q,\epsilon)\Gamma(3-q,\epsilon)}{\Gamma(2-q,\epsilon)^2} \quad \text{[S26]}$$

By using Eq. (S2) and (S5), we obtain exact results for S and S_R in terms of Z:

$$S = \frac{\Gamma(1-q,\epsilon(1+\alpha_+ Z))}{\Gamma(1-q,\epsilon)(1+\alpha_+ Z)^{1-q}} \quad \text{[S27]}$$

$$S_R = \frac{\Gamma(\nu,\epsilon(1+\alpha_+ Z))}{\Gamma(\nu,\epsilon)(1+\alpha_+ Z)^{\nu}} \quad \text{[S28]}$$

Here $\nu = 2 + \chi - q$. The resulting function $R_e/R_0 = S_R(S)$ is shown in Fig. S1 for several values of the exponent q.

For $\chi = 0$, the overall function $S_R(S) = S_e(S)$ can be very well fitted by an empirical analytic formula that depends only on $\lambda_0 = 1 + CV_\alpha^2$ and an additional shape parameter $\Delta_\lambda = CV_\alpha(\gamma_\alpha - 2CV_\alpha)$:

$$S_e \approx \frac{S}{(1 + \Delta_\lambda(1-S))^{(\lambda_0 - 1)/\Delta_\lambda}} \quad \text{[S29]}$$

According to Eq. (S19), this function completely defines behavior of S_R in both limits of the weak and strong correlation regimes :

$$S_R \approx \frac{(1 + (\Delta_\chi - 1)(1-S))\, S}{(1 + \Delta_\lambda(1-S))^{(\lambda - \Delta_\chi)/\Delta_\lambda}} \quad \text{[S30]}$$

Here $\Delta_\chi = (\Delta_\lambda + 1)/\lambda_0$, and $\lambda = \lambda_1$ for $\chi = 1$. For $\chi = 0$, δ_χ has to be set to 1.

Log-normal distribution. The log-normal distribution is a very natural candidate to describe statistics of α. It universally emerges for multiplicative random processes. Transmission of an infection involves a complex chain of random events, both social and biological, which can be conceptualized as such multiplicative process. For instance, it may depend on how likely a given person would be involved in a potential superspreading event, how likely that person would have a close contact with a potential infector, what would be the duration of their contact, how effective the individual immune system is in preventing and suppressing the infection.

For the log-normal distribution, the initial drop in R_e according to Eqs. (10), is noticeably faster than for a gamma distribution: $\lambda = (1 + CV_\alpha^2)(1 + \chi CV_\alpha^2)$. However, the initial linear regime is also much narrower. Figure S1 shows the dependence $R_e(S)$ for the log-normal distribution alongside with the above results for gamma and power law distributions computed for the same values of CV (specifically, $CV_\alpha^2 = 2$). As one can see from these plots, despite a stronger effect of heterogeneity at the early stage, the curves generated by log-normal distribution approach $R_e = 0$ significantly slower than those corresponding to the gamma distribution. Note that the overall behavior of $R_e(S)$ generated by the log-normal distribution closely matches the one obtained for the power law distribution with a certain scaling exponent q. This exponent would depend on CV and should approach 1 in the limit of sufficiently wide distribution when the log-normal pdf asymptotically approaches a power law $1/\alpha$ with upper and lower cut-offs.

Final Size of Epidemic. Here we derive a simple result for the final size of epidemic in a population with a persistent heterogeneity. To do this, we integrate Eq. (S3) over time t, assuming no mitigation, $\mu(t) = 1$. This yields a relation $Z_\infty = \int_0^\infty R_e(t)J(t)dt = \int_{S_\infty}^1 R_e(S)dS/S_e(S)$ for the final value of Z when the epidemic has run its course, and this in turn can conveniently be expressed in terms of the fraction of the susceptible population, S_∞:

$$S_\infty = M_\alpha\left(-\int_{S_\infty}^1 \frac{R_e(S)dS}{S_e(S)}\right) \quad \text{[S31]}$$

This equation is valid for an arbitrary distribution of α, arbitrary correlation between susceptibility and infectivity, and for any statistics of the generation interval. This result can be also obtained as a solution to a general integral equation derived in Ref. (1) for the well-mixed case. Eq. S31 combines and generalizes several well-known results: (i) in the weak correlation limit ($R_\alpha = R_0$), when the integral in the r.h.s. is equal to $R_0(1 - S_\infty)$, Eq.(S31) reproduces results of Refs. (1–4), (ii) in the opposite limit of a strong correlation ($R_\alpha \sim \alpha$), the integration gives $R_0(1 - S_e(S_\infty))/\langle\alpha^2\rangle$, and one recovers the result for the FSE on a network (1, 5, 6).

For the case of gamma-distributed persistent susceptibility Eq. (S31), gives:

$$S_\infty = \left(1 + \frac{R_0\eta\left(1 - S_\infty^{\lambda-\eta}\right)}{\lambda - \eta}\right)^{-1/\eta} \quad \text{[S32]}$$

It should be emphasized however that this result is of limited relevance to more realistic situations. Even if one assumes no government-imposed mitigation or societal response to the epidemic, the case of fully persistent heterogeneity is just an approximation. As we demonstrate in our paper, short-term correlations of time-dependent individual susceptibilities and infectivities lead to transient stabilization of a fast-pacing epidemic. Because of this effect, Eq.(S31)-Eq.(S32) should be interpreted as an estimate of the size of the first wave rather than the actual FSE.

Path-integral theory of epidemic with time-dependent heterogeneity. Here we present a generalization of the theory developed in the previous section that incorporates the effects of time variations of individual susceptibilities and infectivities, as well as temporal correlations between them. Since these fast variations are primarily caused by bursty dynamics of social interactions, and since heterogeneous biological susceptibility appears subdominant in the context of COVID-19, we set $\alpha_b = 1$ for all individuals, so that α has purely social origin. Let $a_i(t) = \alpha_i + \delta a_i(t)$ be the time-dependent susceptibility of a person. Because of the social nature of $a(t)$, one's individual infectivity is also proportional to it at any given time: $\beta_i(t) = R \cdot K(\tau)a_i(t)$. As before, τ is time from infection, $K(\tau)$ is the pdf of generation intervals. Accordingly R is individual reproductive number of an "average" person with social activity $a_i(t) = 1$, in the fully susceptible population. The state of an individual is described by a step function $s_i(t)$ which is 1 as long as the person is susceptible, and turns to 0 at the moment of infection. The time evolution of the epidemic follows a stochastic generalization of (Eqs.(1)-(2):

$$\mathrm{E}\left[\dot{s}_i(t)\right] = -a_i(t)s_i(t-0)J(t) \qquad \text{[S33]}$$

$$J(t) = -\int_0^\infty R \cdot K(\tau)\overline{a_i(t)\dot{s}_i(t-\tau)}d\tau \qquad \text{[S34]}$$

Here bar $\overline{\ldots}$ represents averaging over individual members of population (indexed by i), in contrast with $\langle \ldots \rangle$, averaging over all subgroups with various values of persistent heterogeneity α. E[...] stands for expected value.

The overall quasi-homogeneous description given by Eqs.S3),(S4), remains valid. It is obtained by averaging Eqs. (S33)-(S34) over the entire population. However, in contrast to the case of persistent heterogeneity, variables $S(t)$, $S_e(t)$ and R_e are no longer connected to each other by a simple functional relationship. To relate them we first note that the average probability that an individual is still susceptible at time t is given by $E[s_i(t)] = \exp\left(-\int_{-\infty}^{t} J(t')a_i(t')dt'\right)$. Therefore,

$$S(t, [J(t')]) \equiv \overline{s_i(t)} = \overline{\exp\left[-\int_{-\infty}^{t} J(t')a_i(t')dt'\right]} \qquad \text{[S35]}$$

In other words, S becomes a functional over the set of all possible epidemic trajectories $J(t)$. It still has the structure of a moment generating function for the field $a_i(t)$, and thus is a direct analogue of the partition function broadly used in statistical physics, stochastic calculus, and field theory. The specific form of this functional depends on probabilities assigned to different individual trajectories $a_i(t)$. As a natural generalization of the case of persistent heterogeneity, $S_e(t)$ and $R_e(t)$ can be obtained as, respectively, the first and the second variations of the functional S over $J(t)$:

$$S_e(t) = \overline{a_i(t)s_i(t)} = -\frac{\delta S(t, [J(t')])}{\delta J(t)} \qquad \text{[S36]}$$

$$R_e(t) = R\overline{a_i^2(t)s_i(t)} = R\frac{\delta^2 S(t, [J(t')])}{\delta J(t)^2} \qquad \text{[S37]}$$

For the sake of simplicity, in deriving Eq.(S37) we assumed $\alpha_i(t)$ to be smoothed over the timescale of a single generation interval. As a result, $\int_0^\infty \overline{a_i(t)a_i(t-\tau)}K(\tau)d\tau \approx \overline{a_i^2(t)}$. By applying Eq.(S37) to the initial state of fully susceptible population we obtain the result for R_0:

$$R_0 = R\overline{a_i^2} = R\left(\langle \alpha^2 \rangle + \overline{\delta a_i^2}\right) \qquad \text{[S38]}$$

At the early stages of epidemic $E[s_i(t)] \approx 1 - \int_{-\infty}^{t} a_i(t')J(t')dt'$ for the entire population. After substituting this expression for $s_i(t)$ to Eq.(S37) one obtains a generalization of our previous result for the initial suppression of R_e, Eqs.(9)-(10):

$$R_e(t) \approx R_0\left(1 - \int_0^\infty \Lambda(t,t')J(t-t')dt'\right) \qquad \text{[S39]}$$

$$\Lambda(t,t') = \frac{\overline{\tilde{\alpha}_i^2(t)a_i(t-t')}}{\langle \alpha^2 \rangle + \overline{\delta a_i^2}} = \lambda_\infty + \delta\lambda(t,t') \qquad \text{[S40]}$$

Here $\lambda_\infty = \Lambda(\infty)$ and $\delta\lambda(t,t')$ are the constant and time-dependent contributions to "immunity kernel" $\Lambda(t,t')$ which are discussed in the main text in the context of the definition of λ_{eff}. Note that since the empirical value of λ_{eff} for COVID-19 is relatively large (between 4 and 5), the attack rate at which the TCI state would be achieved is rather low (10%-15%) assuming R_0 between 2 and 3. In this case we are well within the range of our linearized regime. The long-term HIT is determined by a lower value of $\lambda_\infty \simeq 2$. In that case we derived the non-linear dependence of R_e on S without linearization.

To obtain a corrected result for HIT, we assume a very slow progression of the epidemic (e.g. due to a gradual relaxation of the level of mitigation). In this case, any intermediate-term correlations between time dependent variations $\delta\alpha_i(t)$ become negligible, and we largely recover the formalism developed for pure persistent heterogeneity. Belowm we make the same assumption that was used for the estimate of λ_∞ in the main text: $\overline{\delta a_i^2} \sim \alpha_i$. This relationship can be rewritten in terms of $\chi^* = \langle \alpha^2 \rangle / \overline{a_i^2}$, as $\overline{\delta a_i^2} = \chi^* \alpha_i \langle \alpha^2 \rangle$. It leads to the following modification to the result for S_R, Eq(S2):

$$S_R = \frac{R}{R_0}\int \left(\alpha^2 + (1-\chi^*)\overline{a_i^2}\alpha\right) f(\alpha)e^{-\alpha Z}d\alpha = \left(\chi^* \langle \alpha^2 e^{-Z\alpha} \rangle + (1-\chi^*)\langle \alpha e^{-Z\alpha} \rangle\right) \qquad \text{[S41]}$$

Here we used Eq. (S38) for R_0. As one can see, S_R is a linear combination of two earlier results $S_R^{(\chi)}$, for $\chi = 0$ and 1, respectively (both are given by Eq. (S19)):

$$S_R = \chi^* S_R^{(1)} + (1-\chi^*) S_R^{(0)} \tag{S42}$$

For the case of gamma-distributed α, this leads to interpolative result

$$S_R = \chi^* S^{1+\eta} + (1-\chi^*) S^{1+2\eta} \approx S^{\lambda_\infty} \tag{S43}$$

here $\lambda_\infty = 1 + (1+\chi^*)\eta$ is long-term immunity factor obtained in the main text within linearized approximation for $R_e(S)$.

A more detailed derivation of these and other results is at Ref. (7).

Age-of-infection model used to simulate the epidemic dynamics in NYC and Chicago. In our simulations of the COVID-19 epidemic dynamics in Chicago and NYC shown in Fig. 4 we used the age-of-infection model we previously developed for the state of Illinois (8). The daily incidence (i.e. the daily number of newly-infected individuals per capita) $j(t)$, determines the dynamics of susceptible individuals according to

$$\frac{dS(t)}{dt} = -j(t). \tag{S44}$$

The incidence itself follows the renewal equation,

$$j(t) = R_e(t) \int_0^\infty d\tau\, K(\tau) j(t-\tau). \tag{S45}$$

Here, $R_e(t)$ is time-dependent effective reproduction number, $K(\tau)$ is the probability density function (PDF) of generation intervals. We parameterize the effective reproduction number $R_e(t)$ according to Eq. 14

$$R_e(t) = R_0 \mu(t) S(t). \tag{S46}$$

where R_0 is the basic reproduction number, $\mu(t)$ a mitigation factor, $S(t)$ is susceptible population fraction.

For both NYC and Chicago, we have access to reliable data (9–12) describing time series of the following variables

- $H(t)$, the total number of hospitalized (but not critical) patients
- $C(t)$, the number of critically ill patients currently in ICU beds
- $D(t)$, the cumulative number of daily deaths.

In our age-of-infection model changes in these variables are described by daily flux variables:

- $\sigma(t)$, the number of infected individuals who become symptomatic
- $h(t)$, the number of daily admissions to all hospitals
- $r(t)$, the daily number of patients discharged from all hospitals
- $c(t)$, the daily number of patients transferred from the main floor of a hospital to its ICU
- $v(t)$, the daily number of patients transferred from the ICU to the main floor of a hospital, and
- $d(t)$, the daily number of deaths in ICU rooms.

Figure S4 schematically depicts the topology of our model along with the names of all flux and cumulative variables. The dynamics of any flux variable $y(t)$ defined above may be obtained from the variable $x(t)$ directly preceding it in the chain of events shown in Fig. S4:

$$y(t) = p_y \int_0^\infty d\tau\, K_y(\tau) x(t-\tau). \tag{S47}$$

Here, p_y is the proportion of individuals undergoing the transition $x \to y$ with time delays distributed according to a probability density function $K_y(t)$.

We fix the generation interval mean and standard deviation to 4 and 3.25 days respectively (13, 14), while our incubation time distribution has fixed mean 5.5 days and a standard deviation of 2 days (15, 16). We calibrate the remaining delay (τ_x) and fraction (p_x) parameters shown in Figure S4 to data downloaded from (9–12) by sampling over the high-dimensional model parameter space using a Markov chain Monte Carlo (MCMC) approach as described in details in Ref. (8). This procedure allowed us to determine the time evolution of $R_e(t)$ and $S(t)$ in NYC and Chicago, which was used in Fig. 3. Figure S2 shows the $R_e(t)$ divided by the mobility factor calculated from Google community mobility report, Ref. (17). We use the average

of Retail, Grocery, Transit, and Workplaces categories. For NYC we use the average mobility of its five counties: New York county, Bronx county, Kings county, Richmond county, and Queens county, weighted by their population fractions.

To construct Fig. 4 we modified our simulations by replacing Eqs. (S44), (S45) with their quasi-homogeneous generalizations Eqs. (S4), (S3) and setting $S_R = S^\lambda$ and $S_e = S^{1+\eta}$, where $\eta = (\lambda - 1)/2$. After calibrating this model on data up to June 10, 2020 we predicted the effect of relaxing the mitigation factor $\mu(t)$ back to 1 on June 15, 2020. The results of these simulations are shown in Fig. 4. Figs. S5 (NYC) and S6 (Chicago) show our predictions along with 95% confidence intervals caused by parameter uncertainty. Our predictions go up to early September 2020 at which point confidence intervals become too wide.

References

1. Miller JC (2012) A note on the derivation of epidemic final sizes. *Bulletin of mathematical biology* 74(9):2125–2141.
2. Novozhilov AS (2008) On the spread of epidemics in a closed heterogeneous population. *Mathematical Biosciences* 215(2):177–185.
3. Katriel G (2012) The size of epidemics in populations with heterogeneous susceptibility. *Journal of Mathematical Biology* 65(2):237–262.
4. Ball F (1985) Deterministic and stochastic epidemics with several kinds of susceptibles. *Advances in Applied Probability* 17(1):1–22.
5. Newman MEJ (2002) Spread of epidemic disease on networks. *Physical Review E* 66(1):016128.
6. Moreno Y, Pastor-Satorras R, Vespignani A (2002) Epidemic outbreaks in complex heterogeneous networks. *European Physical Journal B* 26(4):521–529.
7. Tkachenko AV, et al. (2021) Stochastic social behavior coupled to covid-19 dynamics leads to waves, plateaus and an endemic state. *medRxiv* 2021.01.28.21250701.
8. Wong GN, et al. (2020) Modeling covid-19 dynamics in illinois under non-pharmaceutical interventions. *Physical Review X* 10.
9. (2020) Data were downloaded from https://www.dph.illinois.gov/covid19/covid19-statistics.
10. (2020) Data from https://github.com/thecityny/covid-19-nyc-data.
11. (2020) Data originally due to https://www.thecity.nyc/.
12. (2020) Data from https://github.com/nychealth/coronavirus-data.
13. Nishiura H, Linton NM, Akhmetzhanov AR (2020) Serial interval of novel coronavirus (COVID-19) infections. *International Journal of Infectious Diseases* 93:284–286.
14. Du Z, et al. (2020) Serial Interval of COVID-19 among Publicly Reported Confirmed Cases. *Emerg Infect Dis* 26(6):2020.02.19.20025452.
15. Lauer SA, et al. (2020) The Incubation Period of Coronavirus Disease 2019 (COVID-19) From Publicly Reported Confirmed Cases: Estimation and Application. *Annals of Internal Medicine* 172(9):577.
16. Linton NM, et al. (2020) Incubation Period and Other Epidemiological Characteristics of 2019 Novel Coronavirus Infections with Right Truncation: A Statistical Analysis of Publicly Available Case Data. *Journal of Clinical Medicine* 9(2):538.
17. (2020) https://www.blog.google/technology/health/covid-19-community-mobility-reports?hl=en.
18. Unwin HJT, et al. (2020) Report 23: State-level tracking of COVID-19 in the United States WHO Collaborating Centre for Infectious Disease Modelling MRC Centre for Global Infectious Disease Analytics.

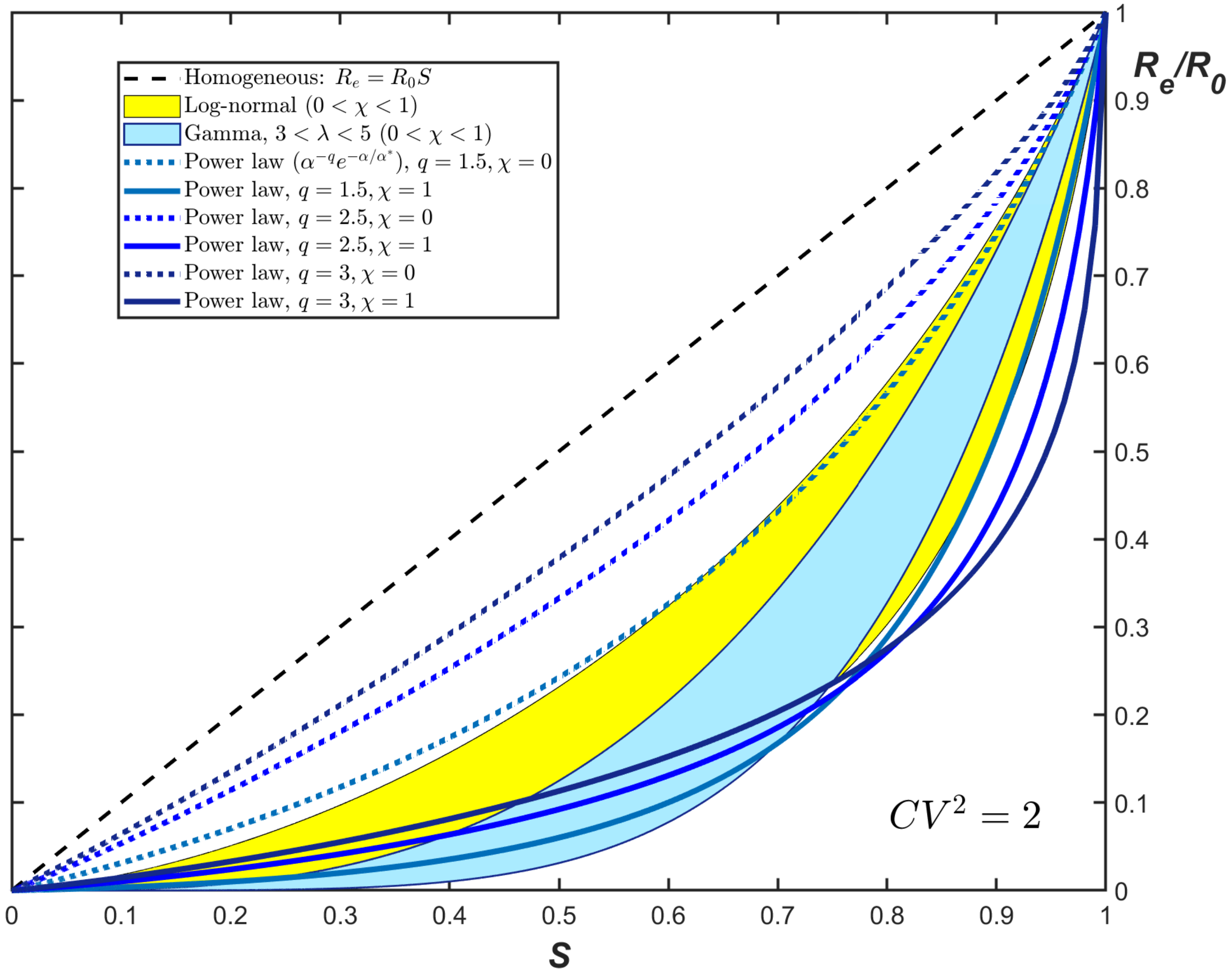

Fig. S1. R_e/R_0 vs S dependence for three different families of probability distribution $f(\alpha)$: Gamma (light blue), truncated power law (dashed lines), and log-normal (yellow). Different curves correspond to the same value of the coefficient of variation $CV_\alpha^2 = 2$, and two limiting values (0 and 1) of the correlation parameter χ.

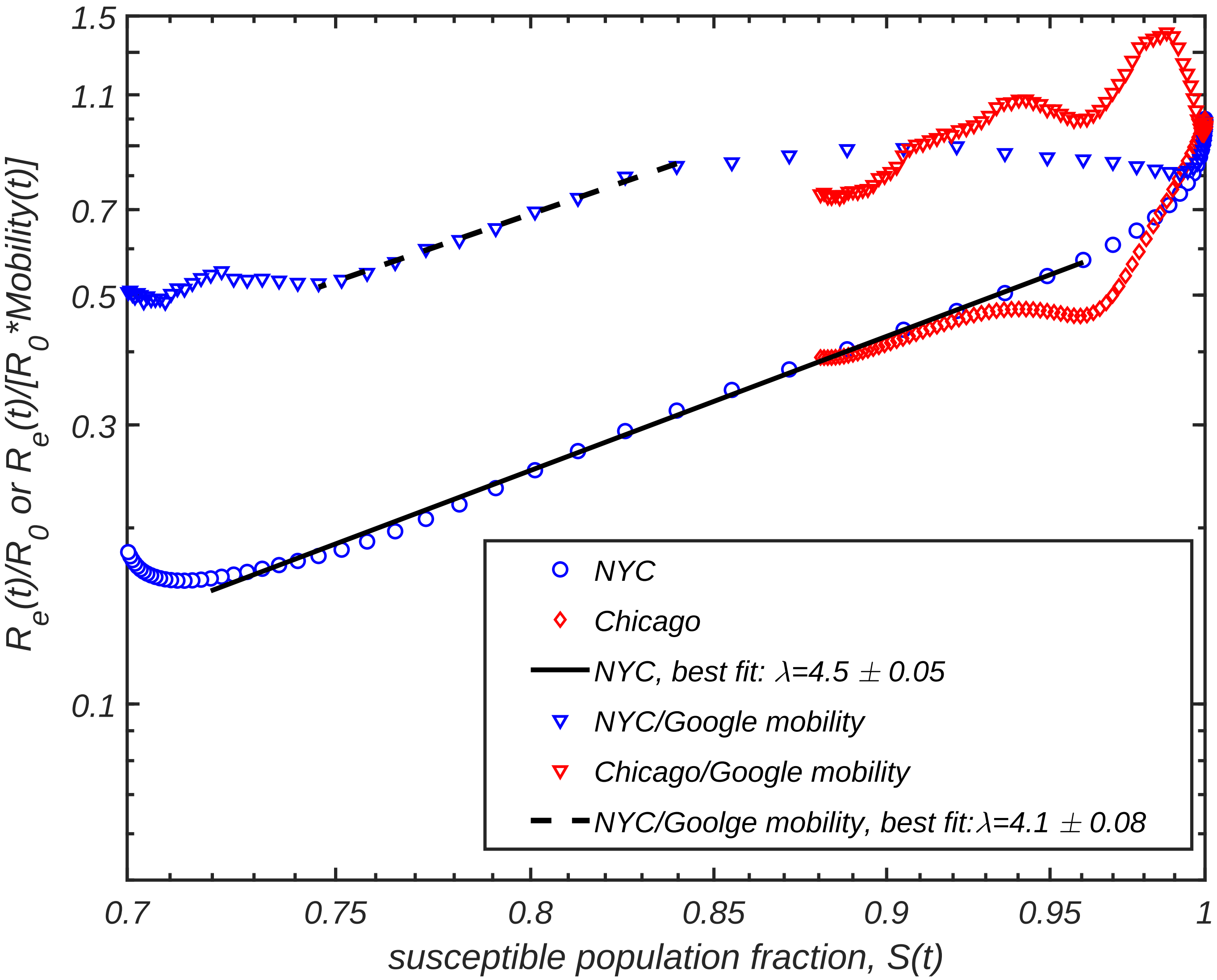

Fig. S2. Exploration of effect of mobility on data presented in Figure 3(A). Triangles represent data points for NYC and Chicago with $R_e(t)/R_0$ corrected by a mobility factor calculated from Google community mobility report, Ref. (17). We compute the mobility for NYC using average mobility of its five counties: New York county, Bronx county, Kings county, Richmond county, and Queens county, weighted by their population fraction.

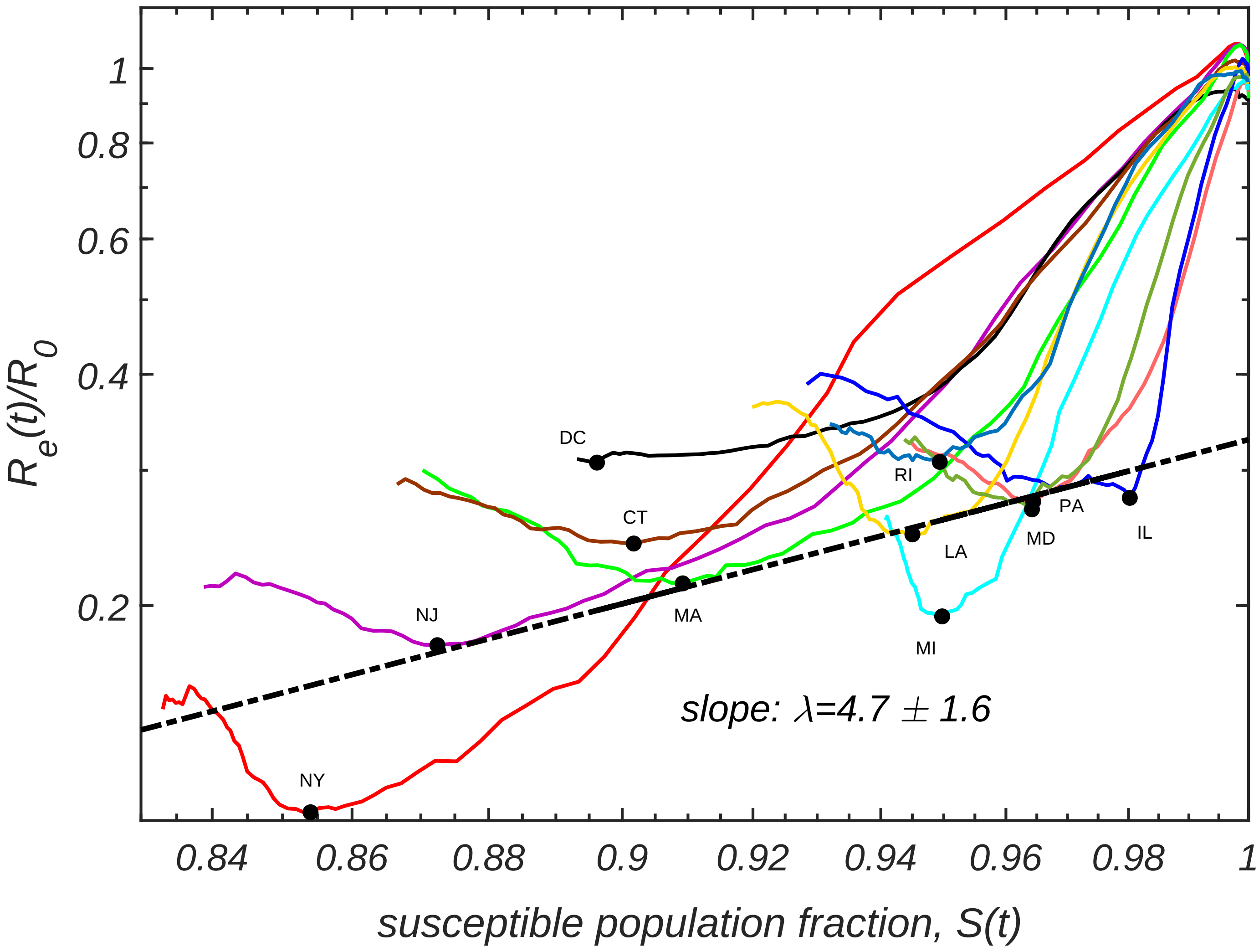

Fig. S3. Time progressions of $Re(t)/R_0$ and $S(t)$ for the hardest-hit US states and DC, as reported in Ref. (18). Black dots correspond to absolute minima of transmission and population susceptible fractions. The dashed line with slope $\lambda = 4.7 \pm 1.6$ is the best power law fit through these black dots.

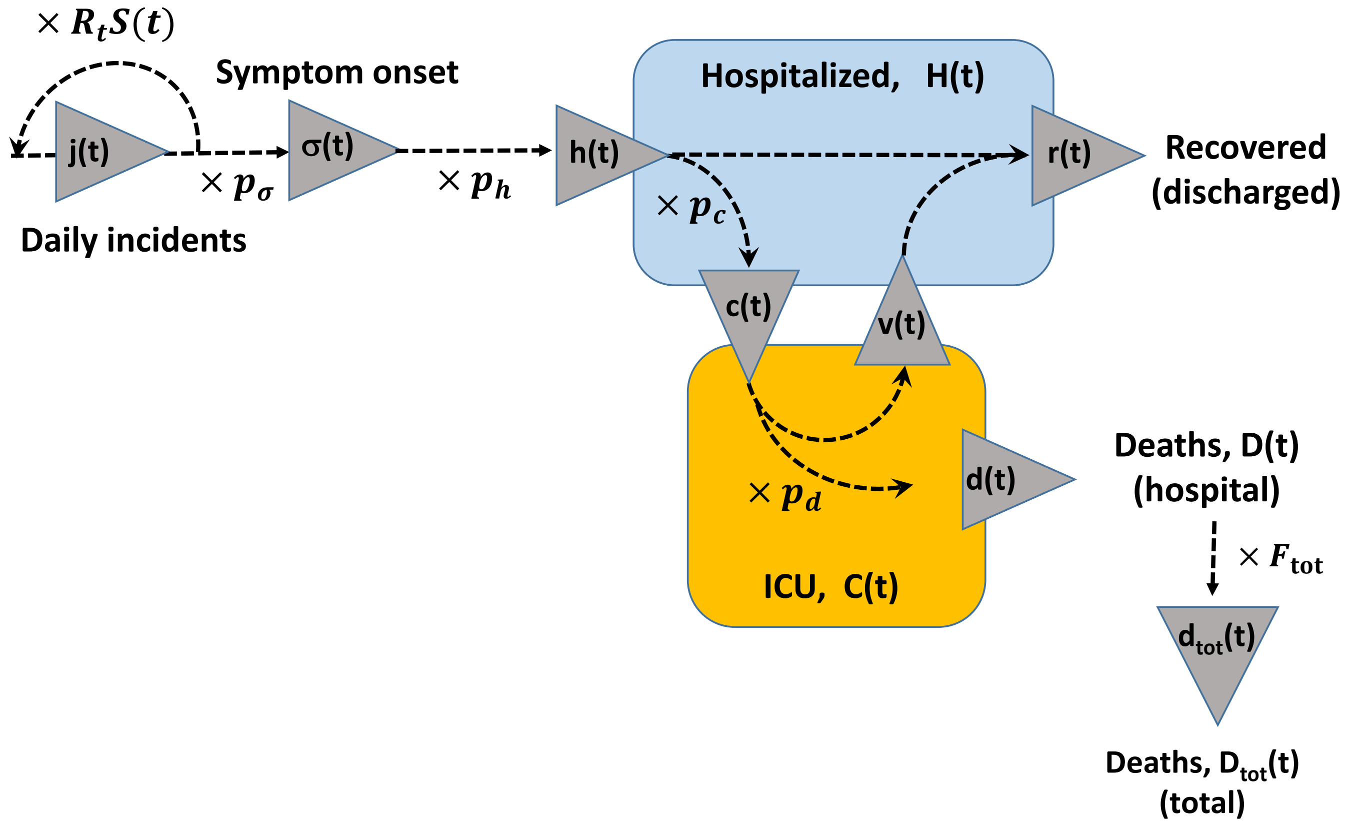

Fig. S4. The topology of our model along with the names of all flux and state variables: the daily incidence, $j_i(t)$; the daily number of newly symptomatic individuals, $\sigma_i(t)$; the number of daily admissions to all hospitals, $h_i(t)$; the daily number of patients discharged from all hospitals, $r_i(t)$; the daily number of patients transferred from the main floor of a hospital to its ICU, $c_i(t)$; the daily number of patients transferred from the ICU to the main floor of a hospital, $v_i(t)$; the daily number of deaths in hospitals, $d_i(t)$; and the daily number of deaths in and out of hospitals, $d_{\mathrm{tot},i}(t)$. State variables are: the total number of occupied hospital beds (main floor) $H_i(t)$, and the total number of occupied ICU beds $C_i(t)$. The other parameters of the model are the fractions of infected individuals who ever become symptomatic, $p_{\sigma,i}$; the fraction of symptomatic individuals who are ever hospitalized, $p_{h,i}$; the fraction of hospital patients who ever get to ICU, $p_{c,i}$; and the fraction of ICU patients who will ultimately die $p_{d,i}$; and the multiplier, F_{tot} that converts between hospital deaths and all deaths in the state, including those outside of the hospital system. For the sake of legibility, we suppress age-group indices in the diagram.

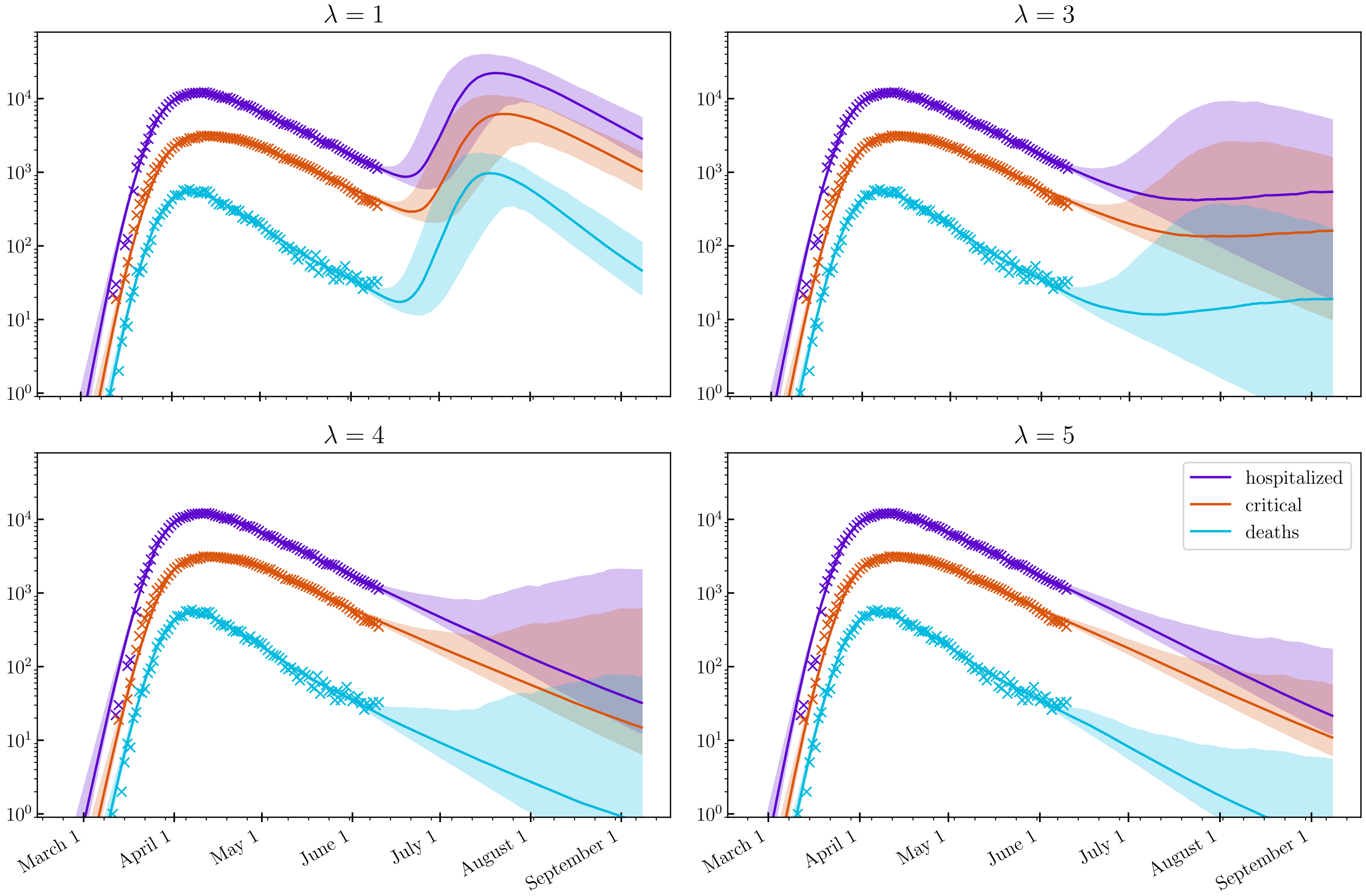

Fig. S5. Hospitalization, ICU occupancy and daily deaths in NYC modeled under hypothetical scenario when any mitigation is completely eliminated as of Jun 15 2020, for various values of λ. Model described in Ref. (8) is calibrated on data from Ref.(9), up to June 10, 2020 (shown as crosses). 95% confidence intervals are indicated.

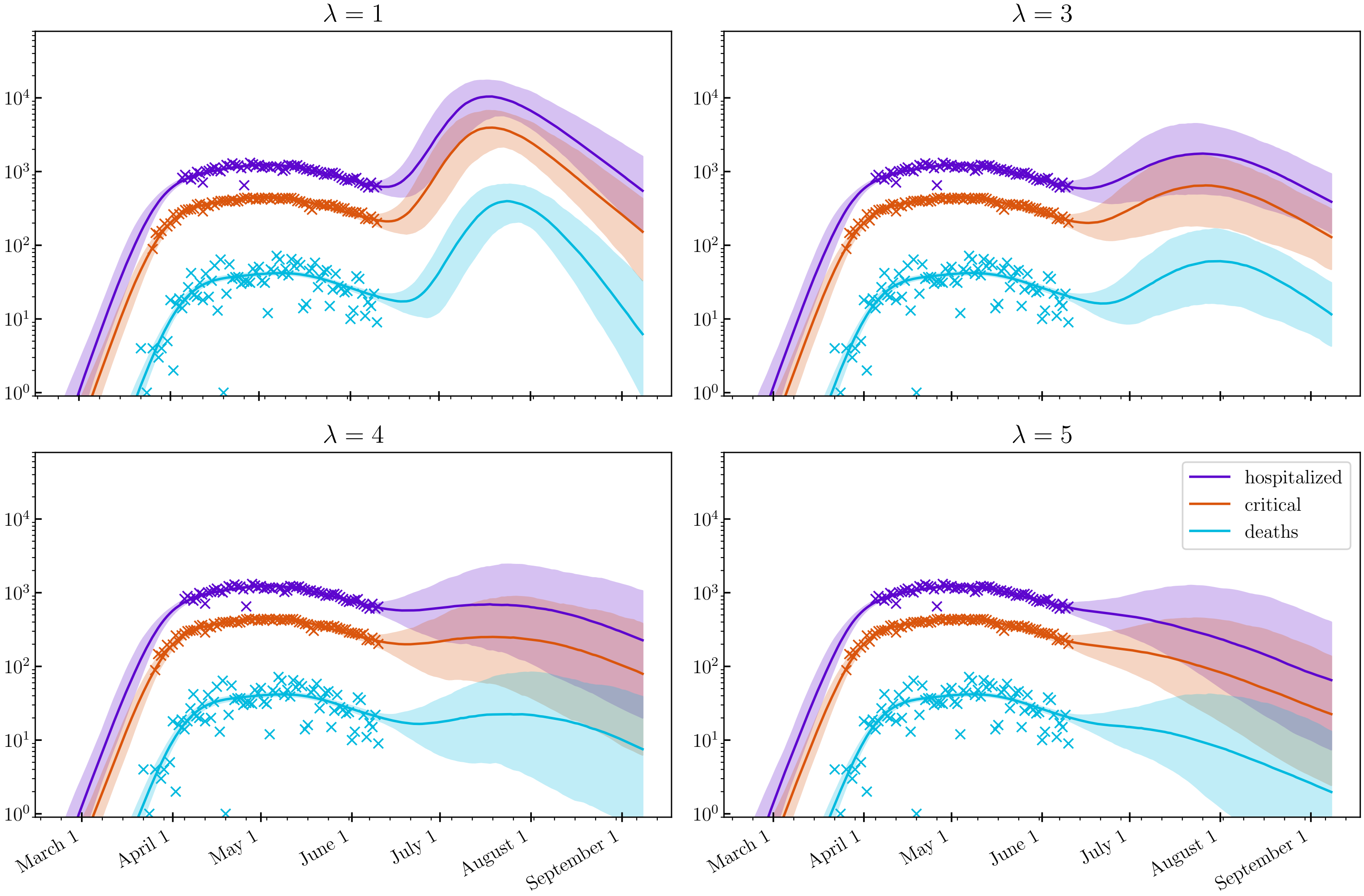

Fig. S6. Hospitalization, ICU occupancy and daily deaths in Chicago modeled under hypothetical scenario when any mitigation is completely eliminated as of Jun 15 2020, for various values of λ. Model described in Ref. (8) is calibrated on data from Ref.(9), up to June 10, 2020 (shown as crosses). 95% confidence intervals are indicated.
